# Gut microbiome changes in overweight male adults following bowel preparation

**DOI:** 10.1186/s12864-018-5285-6

**Published:** 2018-12-31

**Authors:** Hui-Mei Chen, Chung-Chu Chen, Chien-Chi Chen, Shen-Chih Wang, Chun-Lin Wang, Chien-Hsun Huang, Jong-Shian Liou, Ta-Wei Liu, Hwei-Ling Peng, Feng-Mao Lin, Chia-Yuan Liu, Shun-Long Weng, Chieh-Jen Cheng, Yi-Fang Hung, Chii-Cherng Liao, Hsien-Da Huang

**Affiliations:** 1 Institute of Bioinformatics and Systems Biology, National Chiao Tung University, Hsinchu, 300 Taiwan; 2 Division of Hepatology and Gastroenterology, Department of Internal Medicine, MacKay Memorial Hospital, Hsinchu, 300 Taiwan; 3 Teaching Center of Natural Science, Minghsin University of Science and Technology, Hsinchu, 300 Taiwan; 4 Food Industry Research and Development Institute, Hsinchu, 300 Taiwan; 5 Department of Biological Science and Technology, National Chiao Tung University, Hsinchu, 300 Taiwan; 6 Departmnet of Anesthesiology, Taipei Veterans General Hospital, Taipei, 112 Taiwan; 7 School of Medicine, National Yang Ming University, Taipei, 112 Taiwan; 8 Division of Gastroenterology, Department of Medicine & Department of Medical Research, MacKay Memorial Hospital, Taipei, 112 Taiwan; 9 Department of Medicine, MacKay Medical College, New Taipei City, 252 Taiwan; 10 Department of Obstetrics and Gynecology, Hsinchu MacKay Memorial Hospital, Hsinchu, 300 Taiwan; 11 MacKay Medicine, Nursing and Management College, Taipei, 112 Taiwan; 12 School of Science and Engineering, The Chinese University of Hong Kong, Guangdong Province, Shenzhen, 518172 China; 13 Warshel Institute for Computational Biology, The Chinese University of Hong Kong, Guangdong Province, Shenzhen, 518172 China

**Keywords:** Gut microbiome, Overweight, Bowel preparation, High-throughput sequencing, *Bacteroides*, *Preovotella*

## Abstract

**Background:**

Human gut microbiome has an essential role in human health and disease. Although the major dominant microbiota within individuals have been reported, the change of gut microbiome caused by external factors, such as antibiotic use and bowel cleansing, remains unclear. We conducted this study to investigate the change of gut microbiome in overweight male adults after bowel preparation, where none of the participants had been diagnosed with any systemic diseases.

**Methods:**

A total of 20 overweight, male Taiwanese adults were recruited, and all participants were omnivorous. The participants provided fecal samples and blood samples at three time points: prior to bowel preparation, 7 days after colonoscopy, and 28 days after colonoscopy. The microbiota composition in fecal samples was analyzed using 16S ribosome RNA gene amplicon sequencing.

**Results:**

Our results demonstrated that the relative abundance of the most dominant bacteria hardly changed from prior to bowel preparation to 28 days after colonoscopy. Using the ratio of *Prevotella* to the sum of *Prevotella* and *Bacteroides* in the fecal samples at baseline, the participants were separated into two groups. The fecal samples of the Type 1 group was *Bacteroides-*dominant, and that of the Type 2 group was *Prevotella*-dominant with a noticeable presence *Bacteroides*. *Bulleidia* appears more in the Type 1 fecal samples, while *Akkermensia* appears more in the Type 2 fecal samples. Of each type, the gut microbial diversity differed slightly among the three collection times. Additionally, the Type 2 fecal microbiota was temporarily susceptible to bowel cleansing. Predictive functional analysis of microbial community reveals that their activities for the mineral absorption metabolism and arachidonic acid metabolism differed significantly between the two types. Depending on their fecal type, the variance of triglycerides and C-reactive protein also differed between the two types of participants.

**Conclusions:**

Depending upon the fecal type, the microbial diversity and the predictive functional modules of microbial community differed significantly after bowel preparation. In addition, blood biochemical markers presented somewhat associated with fecal type. Therefore, our results might provide some insights as to how knowledge of the microbial community could be used to promote health through personalized clinical treatment.

**Electronic supplementary material:**

The online version of this article (10.1186/s12864-018-5285-6) contains supplementary material, which is available to authorized users.

## Background

Commensal human gut microbiota co-evolve with their host in a symbiotic relationship and influence a large number of biological functions, in particular helping digestion and developing the immune system. Advances in both sequencing technologies and bioinformatics tools have greatly improved our knowledge of the role of gut microbiome in human health and disease [1–3]. In recent years, modulation of gut microbiome has been considered one of the methods of improving health. However, the change of gut microbiome caused by external factors, such as antibiotic use and bowel cleansing, remains unclear. The gut microbial composition is strongly correlated with environmental factors such as dietary habit and drug usage [4, 5]. The gut microbiome is also linked to host weight [6, 7]. In Taiwan, over 49.9% of Taiwanese males are either overweight or obese, with a body mass index (BMI) greater than or equal to 25 (kg/m^2^), as reported by the Health Promotion Administration of Taiwan in 2016. Therefore, further understanding of the composition of gut microbiota and the change of gut microbiome within overweight individuals is needed in order to modulate gut microbiome via external strategies such as probiotics or bowel cleansing.

Due to the high risk of metabolic diseases within people who are either overweight or obese [8], the gut microbiome of the obese is of particular interest. In 2005, Ley and her colleagues reported that obesity was associated with the ratio of the abundance of Bacteroidetes and Firmicutes in genetically obese mice [9]. Subsequently, a decreased proportion of Bacteroidetes and an increased proportion of Frimicutes were observed in obesity [10, 11]. Nevertheless, another study, using fluorescent in situ hybridization, did not support this hypothesis [12]. A large population sample study, called Inter99, revealed that some obese people with lower bacterial richness gained more weight over time [13]; and also found that the population could be separated into two groups differing in the number of their gut microbial genes and the gut bacteria richness. Recently, research in Japanese and Chinese populations also revealed that gut microbial composition differed between obese people and lean people [14–16]. Further, a study using metagenomics sequencing found that obesity-associated gut microbial species is linked to changes in circulating metabolites and that the abundance of *Bacteroides thetaiotaomicron* decreased in obese people [17].

The effects of probiotic-based treatment are different from person to person [18]. Until now, the most common probiotics include *Bifodobacteria*, *Lactobacilli*, *Enterococci*, and yeasts; and their effect on human health and disease have been reported [19, 20]. For example, *Lactobacillus rhamnosus* CGMC1.3724 formulation may influence weight loss in obese women [21]; and *Lactobacillus gasseri* SBT2005 may regulate abdominal adiposity in Japanese populations [22, 23]. In contrast, some *Lactobacillus* species including *L. acdophilu*s might lead to weight gain, not loss [24]. Thus, different species might result in diverse effects. Now fecal material transplantation (FMT) has become another treatment option to modify gut microbiome. Beneficial effects of FMT are shown in patients with *Clostridium difficile* infections (CDI) or inflammatory bowel disease (IDB) [25, 26]. In addition, another study showed that insulin sensitivity increases in obese participants with metabolic syndrome after the transfer of intestinal microbiota from lean donors [27]. Additionally, it is noticeable that bowel preparation may change gut microbiome [28–30]. Therefore, it is important to understand the influence of external environmental factors on the variation of gut microbiome.

Inspired by the study of Enterotype and *Prevotella*-ratios [31, 32], and due to ethnic and cultural variance, we focused on omnivore, overweight, male Taiwanese adults. The two main aims of this study are addressed by the following questions: What are the dominant gut microbiota, and to what extent does microbial diversity change after bowel preparation? Which predictive functional modules in fecal community differ most from before bowel preparation to after colonoscopy? Our results might provide some insights as to how knowledge of the change in microbial community could be used to promote health through personalized clinical treatment.

## Methods

### Experimental design and participant selection

**Fig. 1:**
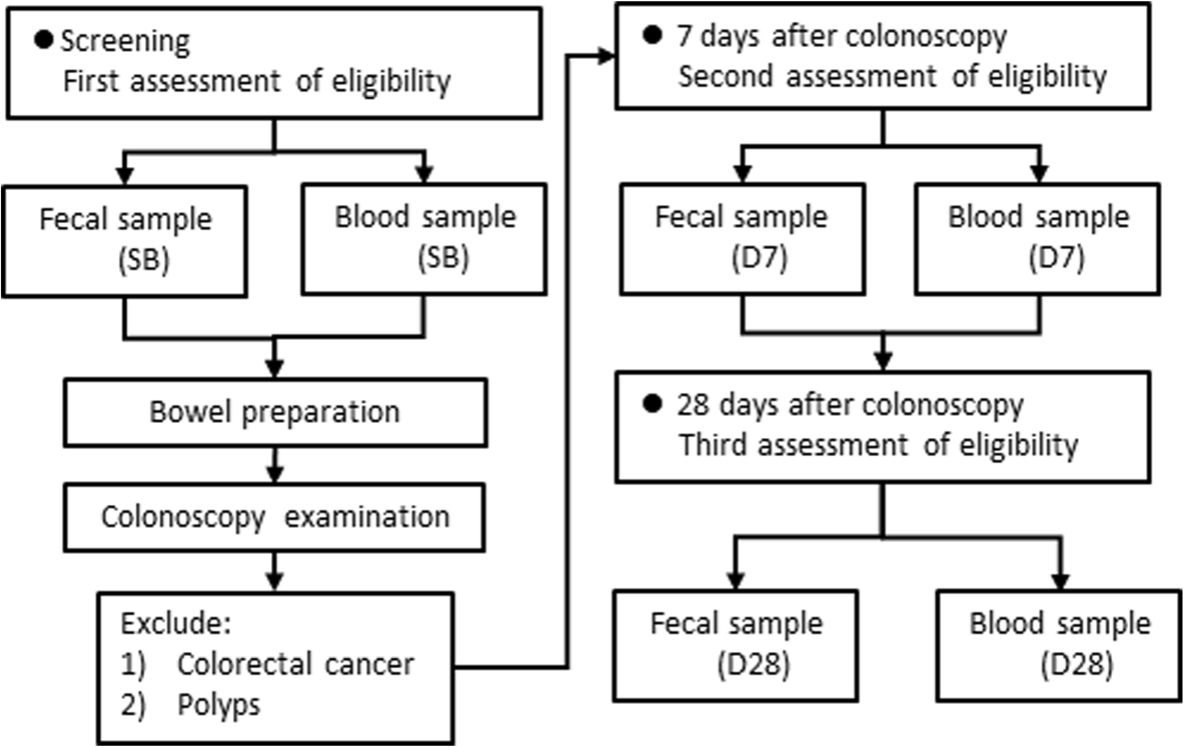
Experimental design. SB represents baseline, which means the first sample collection time prior to bowel preparation. D7 and D28 represent 7 days and 28 days after colonoscopy, respectively. Samples identified with colorectal cancer or polyps, upon inspection of the colonoscopy, were excluded from the analysis

The experimental design is illustrated in Fig. 1. The inclusion criteria of participants were male adults whose age ranged from 20 to 60 years, with body mass index (BMI) equal or greater 25 (kg/m^2^). The exclusion criteria are listed in Additional file 1: Table S1. At screening period, twenty-four overweight male participants were met the entry criteria. During the inspection of colonoscopy, four participants identified as colorectal cancer, or polyps were excluded. Finally, a total of twenty participants completed post-procedure visits for the following analysis.

### Bowel preparation

A low-residue diet that avoids foods containing seeds and other indigestible substances is recommended for three days before the colonoscopy. The split-dose of bowel preparation was administrated by Fleet Phospho-Soda Oral Saline Laxative (sodium phosphate) with water (total 3–4 l). The second dose was administered 4 to 5 h before the planned start of the colonoscopy. All of the patients achieved to the level of good (minimal turbid fluid) or excellent (mucosal detail clearly visible) by Ottawa Bowel Preparation Scale.

### Fecal samples and blood samples

Participants provided fecal samples and blood samples prior to bowel preparation, 7 days after colonoscopy, and 28 days after colonoscopy. Fecal samples were collected by participants at home and were put in refrigerator as well as informed a research nurse to deliver these samples to the central laboratory at Food Industry Research and Development Institute. Each fecal sample was collected using two fecal collection devices: one for 5 g and the other for 15 g. The fecal samples were designed to evaluate the gut microbiota composition as well as fecal calprotectin.

Blood samples were taken after an overnight fast for the determination of plasma lipid profile, fasting plasma glucose (FPG) and immune response. Approximately ~ 14 mL of blood was collected into three independent blood drawing tubes: 3 mL for complete blood count (CBC) of safety lab tests, 3 mL for blood biochemistry of safety lab tests, and 8 mL for inflammatory cytokine analysis. Serum samples were centrifuged and stored at − 80 °C until analysis. Fasting or 2-h glucose, serum alanine aminotransferase (ALT), aspartate aminotransferase (AST), triglycerides (TG), total cholesterol (TC), high-density lipoprotein (HDL) cholesterol, and low-density lipoprotein (LDL) cholesterol were measured using an autoanalyser (Beckman Coulter AU5800). Serum IL-6 and TNF-α were measured using a MILLIPLEX MAG Human Adipokine Magnetic Bead Panel (Millipore) according to the manufacturer’s instructions.

### Bacteria DNA extraction

Fecal samples were stored at − 80 °C prior to the DNA extraction. Total DNA was extracted from fecal material using a modified protocol of the QiaAmp DNA Mini Stool Kit (Qiagen, Hilden, Germany). Briefly, each sample was centrifuged at 13,000 rpm for 2 min, and the resulting bacterial pellet was resuspended in 180 μl of enzyme solution (20 mg/ml lysozyme, 20 mM Tris-HCl [pH 8.0], 2 mM ethylenediaminetetraacetic acid, and 2% sodium dodecyl sulfate). Lysates were incubated at 37 °C for 30 min prior to the addition of 20 μl proteinase K (25 mg/ml) and 200 μl Buffer AL. Each suspension was subsequently incubated at 56 °C for 30 min, and for a further 15 min at 95 °C. The final DNA was eluted with 30 μl of Buffer AE, and stored at − 20 °C for further analysis.

### 16S rRNA gene amplification

The PCR primers F515 (5′-GTGCCAGCMGCCGCGGTAA-3′) and R806 (5′-GGACTACHVGGGTWTCTAAT-3′) were designed to amplify the V4 region of the bacterial 16S ribosomal DNA as described previously [33]. PCR amplification was performed in a 50-μl reaction volume containing 25 μl 2X Taq Master Mix (Thermo Scientific), 0.2 μM of forward and reverse primer, and 20 ng DNA template. The reaction process increased the initial temperature to 95 °C for 5 min, followed by 30 cycles of 95 °C for 30 s, 54 °C for 1 min, and 72 °C for 1 min as well as a final extension of 72 °C for 5 min. Next, amplified products were checked by 2% agarose gel electrophoresis and ethidium bromide staining. Amplicons were purified using the AMPure XP beads (Agencourt) and quantified using the Qubit dsDNA HS Assay Kit (Thermo Fisher Scientific), all according to the respective manufacturers’ instructions. For V4 library preparation, Illumina adapters were attached to the amplicons using the Illumina TruSeq DNA Sample Preparation v2 Kit. Purified libraries were processed for cluster generation and sequencing using the MiSeq system.

### Taxonomy assignment for bacterial 16S rRNA sequences

A pipeline combing PANDAseq [34] and QIIME [1] was used to analyze the raw sequences. The 16S rRNA gene sequences were collected from the National Center for Biotechnology Information Sequence Read Archive in August 2012. Using the default values in QIIME and the taxonomic threshold of 97% in UCLUST [35], high-quality reads were binned into operational taxonomical units (OTUs).

### Statistical analysis

Taxa of the same type were agglomerated at the phylum, class, order, family, and genus levels. The Type 1 and Type 2 groups were classified by a *Prevotella* ratio of the sum of *Bacteroides* and *Prebotella* within the fecal samples at baseline. The microbial diversity was evaluated from two aspects: alpha diversity and beta diversity. In this study, alpha diversity consisted of richness which means the number of genera, and Shannon diversity index, at the genus level. The variances of richness and Shannon diversity index between the two type groups were evaluated by the nonparametric analysis including the Wilcoxon rank sum test and the Kruskal-wallis test. The strength of correlation was measured using the Spearman’s rank correlation coefficient. Beta diversity was evaluated using Phylogeny-based UniFrac analysis [36]. The principal coordinate analysis method was used for visualisation based on data reduction of patterns in an n-dimensional dataset. Six correlation networks of specific genera were built based on the Spearman correlation coefficient. Predictive functional profiling of microbial communities using PICRUSt (phylogenetic investigation of communities by reconstruction of unobserved states) was carried out the 10-base logarithm-transformed data for further analysis [37]. The variances of each predictive functional module and blood biochemical test were evaluated by nonparametric analysis including the Wilcoxon rank sum test and the Kruskal-wallis test. The adjust-*P* values were evaluated by the Bonferroni correction. R software was used for statistical analysis (The R Project for Statistical Computing, Vienna, Austria).

## Results

### Characteristics of the study population

A total of 20 overweight, male Taiwanese adults were recruited. All participants were omnivorous. Their age ranged from 28 to 53 years, with an average of 40.5 years (SD = 7.03); and their body mass index (BMI) ranged from 25.7 to 34.2 (kg/m^2^), with an average BMI of 28.9 (SD = 2.6). All participants did not have systemic diseases such as anemia, hypertension, diabetes mellitus, chronic kidney disease or abnormal liver function. The clinical data and biochemical data were shown in Table 1. Participants provided fecal samples and blood samples at three time points: prior to bowel prep, 7 days after colonoscopy, and 28 days after colonoscopy. For convenience, the three collection time points were denoted as SB, D7, and D28, respectively.

**Table 1:** Characteristics of 20 male samples and biochemical data (Mean values with their standard deviation)

	SB		D7		D28	
	Mean	SD	Mean	SD	Mean	SD
Age (years)	40.50	7.03	40.50	7.03	40.50	7.03
BMI (kg/m^2^)	28.92	2.63	28.80	2.65	29.00	2.65
Hgb (g/dl)	14.83	1.53	14.89	1.60	14.87	1.70
Fasting blood sugar (mg/dl)	98.15	11.86	101.20	12.66	102.15	16.10
SGPT (IU/L)	35.00	17.65	35.50	20.13	36.50	20.25
SGOT (IU/L)	24.20	8.32	24.10	9.26	25.65	10.57
Triglyceride (mg/dl)	182.75	92.24	181.65	93.75	185.65	101.70
Total cholesterol (mg/dl)	212.60	35.95	217.90	39.40	217.25	37.29
HDL cholesterol (mg/dl)	42.20	8.75	41.50	8.34	41.30	7.67
LDL cholesterol (mg/dl)	133.20	30.83	139.45	35.43	139.45	38.57
C-reactive protein (mg/L)	0.34	0.46	0.23	0.22	0.24	0.20
IL-6 (pg/mL)	2.99	3.13	3.83	9.02	2.91	4.07
TNF-alpha (pg/mL)	2.30	7.85	2.85	6.85	2.29	7.05

*SD* standard deviation, *BMI* body mass index, *SB* baseline, *D7* 7 days after colonoscopy, *D28* 28 days after colonoscopy, *Hgb* hemoglobin, *AST* aspartate aminotransaminase, *ALT* alanine aminotransaminase, *HDL* high density lipoprotein, *LDL* low density lipoprotein, *IL-6* Interleukin-6, TNF-alpha, tumor necrosis factor alpha

### *Prevotella*-ratio separated into two groups

**Fig. 2:**
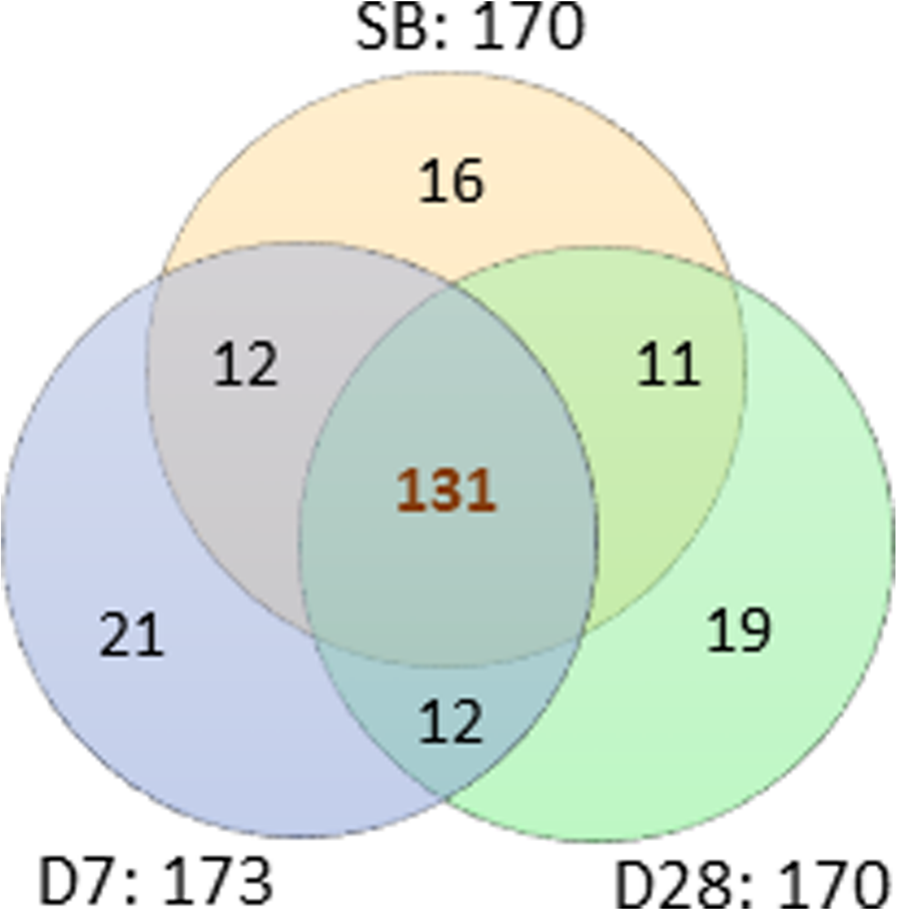
Venn diagram of the number of genera in the three fecal sample groups. SB, D7, and D28 mean the three sample collection time points: prior to bowel preparation; 7 days after colonoscopy; and 28 days after colonoscopy, respectively. The number of genera detected in all three fecal groups is 131

**Fig. 3:**
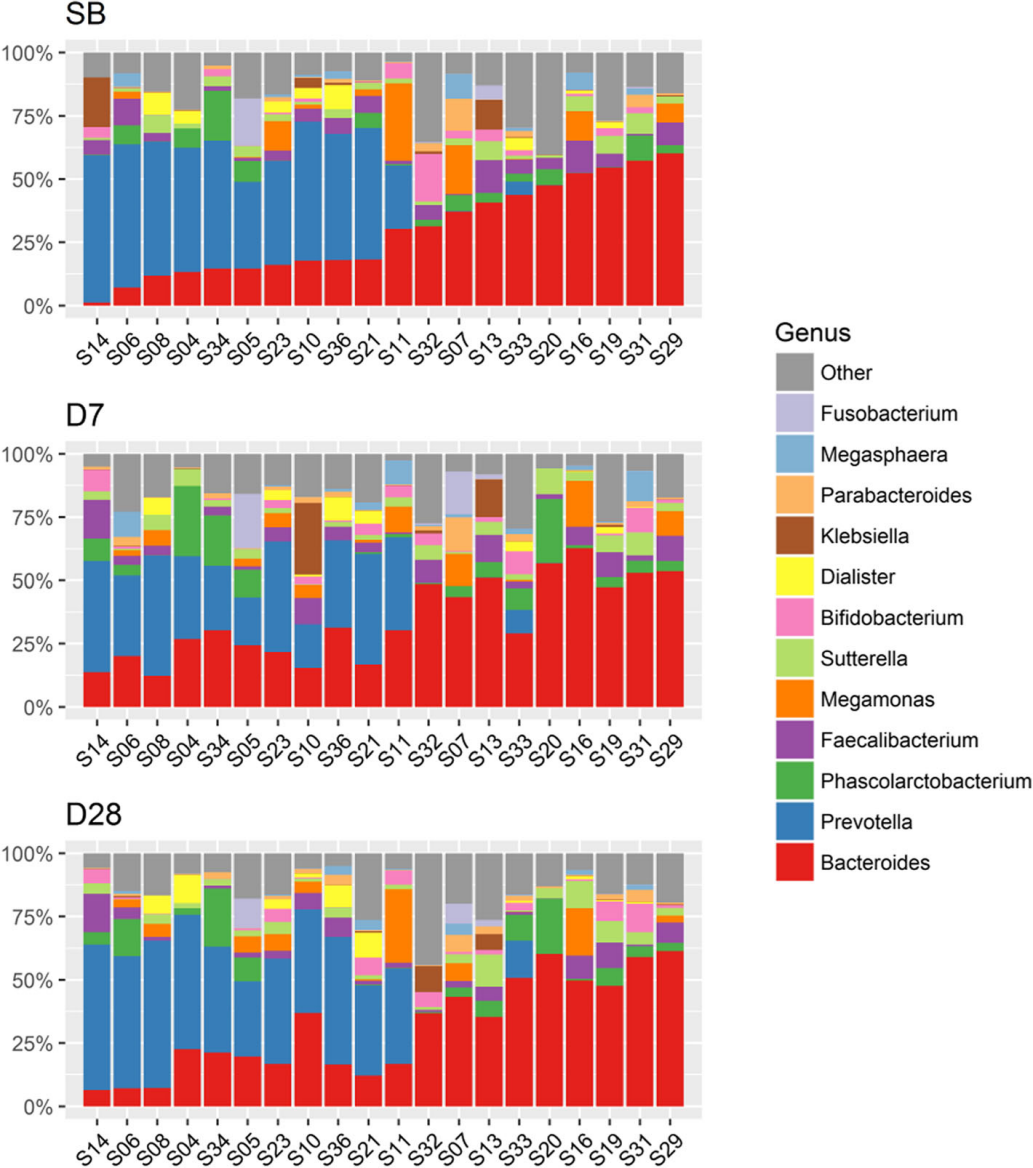
Microbiota composition of each fecal sample over the three collection times. The fecal microbiota composition profiles at genus level are revealed by 16S rRNA amplicon sequencing. Each color represents one bacterial genus. The two most abundant genera within most individuals were *Bacteroides* and *Prevotella*

We analyzed the microbiota composition in fecal samples using 16S rRNA gene amplicon sequencing. The number of genera detected in the three time collections of fecal samples was 131. Of these, 16 genera were detected in every fecal sample collected, regardless of the collection time point (Fig. 2 and Additional file 2: Table S2). According to the results of an average relative abundance in all fecal samples, the most abundant genera were *Bacteroides* (31.7% of all assigned reads) and *Prevotella* (23.8%). The next were *Phascolarctobacterium* (5.5%), *Faecalibacterium* (4.9%), and *Megamonas* (4.1%). Figure 3 depicts fecal microbiota composition at the genus level within individuals. It also reveals that *Bacteroides* and *Prevotella* abundance in the SB fecal sample is essentially restored 28 days after colonoscopy.

By evaluating the ratio of *Prevotella* to the sum of *Prevotella* and *Bacteroides* within individuals, the SB fecal samples was separated into two groups. One group had a *Prevotella*-ratio lower than 0.11, denoted as Type 1; and the other had a *Provetella*-ratio higher than 0.45, denoted as Type 2 (Table 2). The participants will be separated into two groups depending on their SB fecal type. Based on the two group, the results of the analysis will be explained from two aspects: first, comparing the differences of fecal microbiome between types 1 and 2, at each of the three collection times; and second, over the three collection times, comparing the differences of fecal microbiome of each type separately.

**Table 2:** *Prevotella* ratios of the sum of *Bacteroides* and *Prevotella*

		*Prevotella*/(*Bacteroides* + *Prevotella*)		
ID	Type	SB	D7	D28
S029	Type 1	0.0001	0.0003	0.0001
S031	Type 1	0.0001	0.0000	0.0004
S013	Type 1	0.0004	0.0012	0.0005
S032	Type 1	0.0005	0.0004	0.0009
S019	Type 1	0.0006	0.0011	0.0002
S016	Type 1	0.0006	0.0004	0.0002
S020	Type 1	0.0027	0.0024	0.0002
S007	Type 1	0.0033	0.0009	0.0002
S033	Type 1	0.1066	0.2413	0.2253
S011	Type 2	0.4544	0.5484	0.6942
S005	Type 2	0.6990	0.4369	0.6040
S023	Type 2	0.7182	0.6685	0.7139
S036	Type 2	0.7343	0.5240	0.7536
S021	Type 2	0.7405	0.7220	0.7460
S010	Type 2	0.7554	0.5266	0.5256
S034	Type 2	0.7752	0.4563	0.6652
S004	Type 2	0.7872	0.5485	0.7028
S008	Type 2	0.8165	0.7948	0.8897
S006	Type 2	0.8880	0.6126	0.8794
S014	Type 2	0.9802	0.7611	0.8990

### A degree of microbial diversity change in fecal community between the two groups

In this study, the richness of microbial diversity means the number of genera detected within each individual. In the SB fecal samples, the interquartile range of richness was wider in the Type 1 group than in the Type 2 group, but the difference in richness was not statistically significant (Additional file 3: Figure S1A). However, in the D28 fecal samples, the interquartile range of richness was wider in the Type 2 group than the Type 1 group, and the richness was significantly different between the two types (*P* = 0.025, Additional file 3: Figure S1A). As for the Shannon diversity index, there was no statistical difference between the two types at each of the three collection times, but in the SB fecal samples, the interquartile range of Shannon diversity index was wider in the Type 1 group than in the Type 2 group (Additional file 3: Figure S1B and Additional file 4: Table S3). Furthermore, the correlation coefficient between richness and Shannon diversity index of each type became closer from SB to D28 (Additional File 3: Figure S1C).

**Fig. 4:**
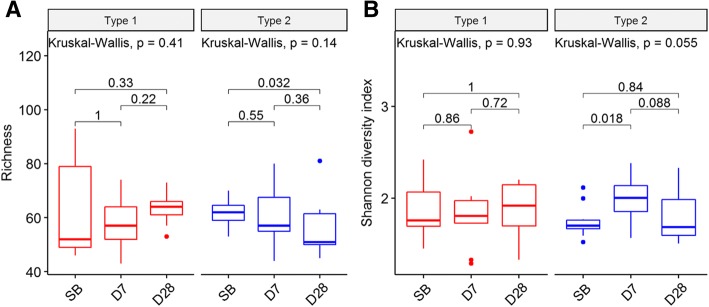
The change of microbial diversity for each type. (**a**) Boxplot of richness. In the Type 1 group, the richness does not show significant differences between any of the collection time points, but the spread of richness decreases over the three collection times. In the Type 2 group, the richness significantly differed between SB and D28 (*P* = 0.032). (**b**) Boxplot of Shannon diversity index. In the Type 1 group, the Shannon diversity index does not show significant differences between any of the collection time points. In the Type 2 group, the Shannon diversity index significantly differs between SB and D7 (*P* = 0.018)

Next, in the Type 1 group, both the richness and Shannon diversity index did not show significant differences between any of the collection time points, but the interquartile range of richness decreased over the three collection times (Fig. 4). Unlike in the Type 1 group, the richness of the Type 2 group significantly differed between SB and D28 (*P* < 0.032, Fig. 4A); and the Shannon diversity index significantly differed between SB and D7 (*P* = 0.018), but not between SB and D28 (Fig. 4B). In addition, the interquartile range of richness in the Type 2 group increased over the three collection times (Fig. 4B).

**Fig. 5:**
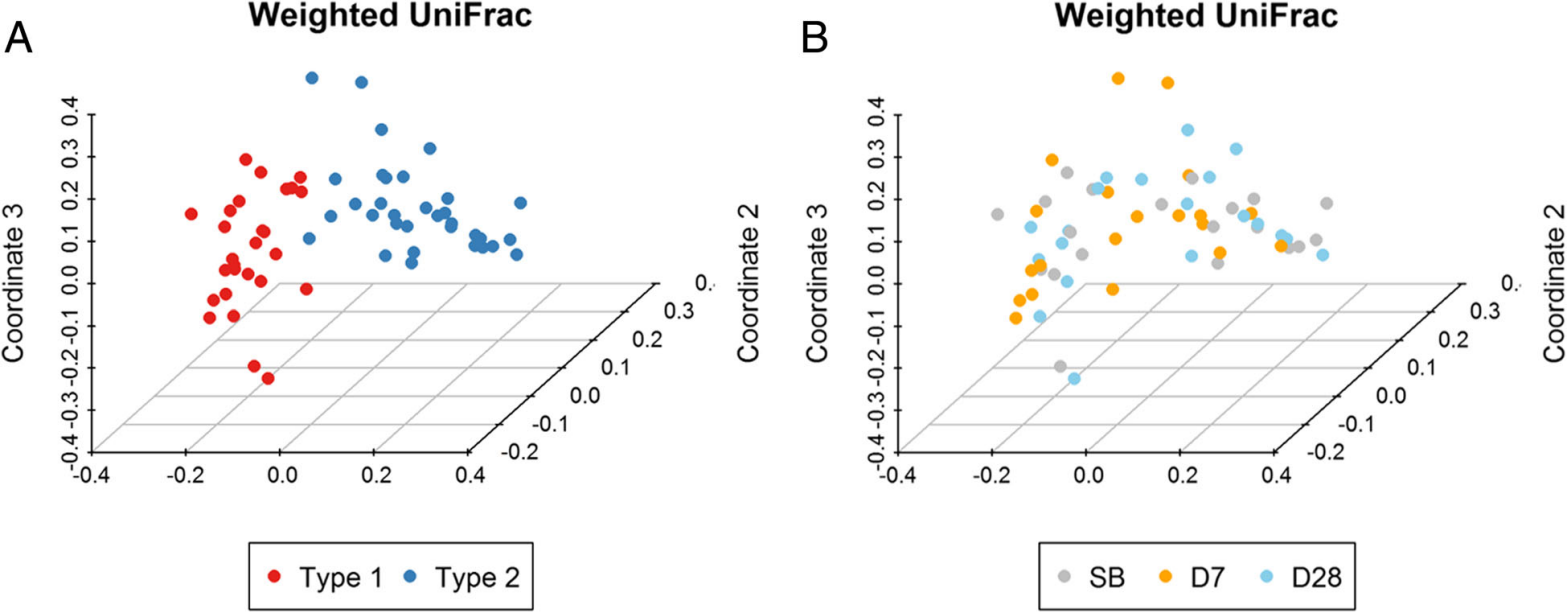
Principal coordinate analysis (PCoA) based on weighted UniFrac distance. Each point represents one fecal sample. (**a**) Red color means the Type 1 group, and blue color means the Type 2 group. The 60 fecal samples were separated into two clusters. (**b**) The three colors represent the three collection sample times. Gray means prior to bowel preparation, denotes as SB; orange means 7 days after colonoscopy, denoted as D7; and blue means 28 days after colonoscopy, denoted as D28. The difference of microbial diversity within individuals did not change remarkably over the three collection times

According to the results of the weighted UniFrac distance, the 60 fecal samples were separated into two clusters. The Type 1 fecal samples formed one cluster in which the most abundant genus was *Bacteroides*; and the other group was formed from the Type 2 fecal samples in which the most abundant genus was either *Prevotella*, *Megomonas*, or *Klebsiella* (Fig. 5A and Additional file 5: Fig. S2). Whereas, regardless of the type, the difference of microbial diversity within individuals based on the weighted UniFrac distance did not change remarkably over the three collection times (Fig. 5B).

### Significant differences in microbiota between the two groups

Among the genera which were detected in at least 50% of the all fecal samples, no genera differed significantly in the Type 1 fecal samples from SB, through D7, to D28. Only four genera: *Bacteroides*, *Prevotella*, *Oscillospira*, *Holdemania* changed significantly in the Type 2 fecal samples from SB to D7 (*P* < 0.05). In addition, at any sample collection time, *Bacteroides* and *Prevotella* differed markedly between the Type 1 and Type 2 groups (*P* < 0.05). The following genera also changed significantly between the Type 1 and Type 2 groups at differing sample collection times: two genera, *Akkermansia* and *Paraprevotella,* at SB; five genera, *Akkermansia*, *Paraprevotella*, *Actinomyces*, *Oscillospira*, and *Fusobacterium* at D7; and 3 genera *Paraprevotella*, *Streptococcus*, and *Enterococcus* at D28.

**Fig. 6:**
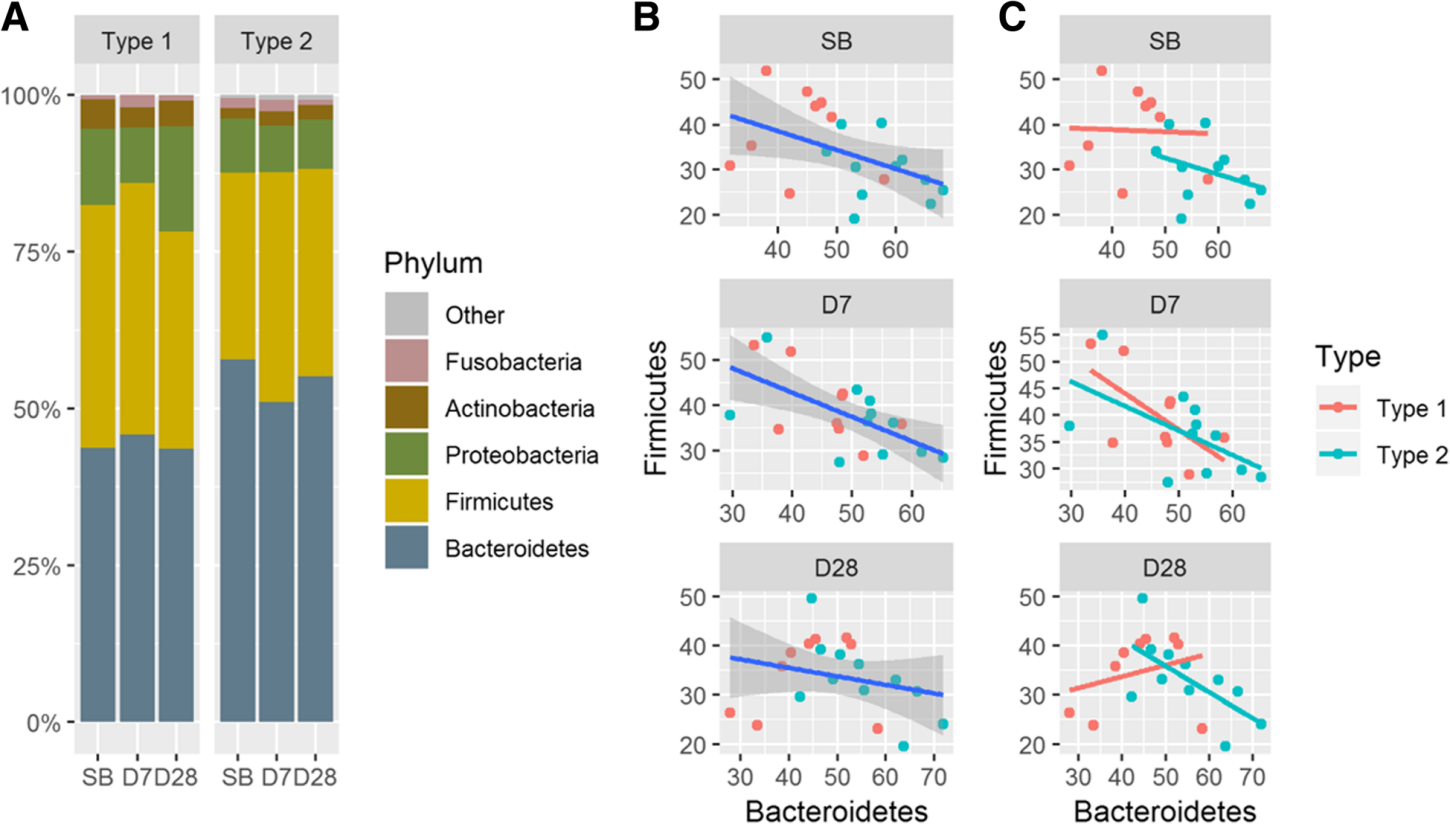
Microbial distribution at phylum level. (**a**) Phylum-level microbial composition of fecal community. Two phyla, Bacteroidetes and Firmicutes, are dominant within most individuals. (**b**) Scatter plots between Bacteroidetes and Firmicutes for each type at the three collection time points. The correlations between Bacteroidetes and Firmicutes at SB and D7 were negative (Spearman ρ = − 0.53 and − 0.45, respectively; *P* < 0.05). (**c**) The correlation between Bacteroidetes and Firmicutes in the Type 1 samples changed markedly from SB to D28 compared with the Type 2 samples

We also found that at the phylum level, the percentage of three phyla, Bacteroidetes, Firmicutes, and Proteobacteria, of each type changed slightly over the three collection times (Fig. 6A). At SB and D7 collection times, the correlations between Bacteroidetes and Firmicutes were negative (Spearman ρ = − 0.53 and − 0.45, respectively; *P* < 0.05) (Fig. 6B). At D28, the correlation between Bacteroidetes and Firmicutes was weakly negative without statistical significance (Spearman ρ = − 0.25; *P* = 0.27) (Fig. 6B). Furthermore, in the Type 1 group, the correlations were 0.00, − 0.40, and 0.28 at SB, D7, and D28, respectively; and in the Type 2 group, the correlations were − 0.35, − 0.58, and − 0.65 at SB, D7, and D28, respectively (Fig. 6C).

### A degree of microbial correlation change after bowel preparation

The correlation networks for 61 genera, which were detected in at least 50% of the fecal samples at a particular collection time, are presented in the supplementary figures (Additional file 6: Figure S3-S5). The strength of correlation was evaluated using Spearman’s correlation coefficient. To further observe the changes of the correlation over the three collection times, the 1830 correlation coefficients were separated into three subintervals [− 1, − 0.5], (− 0.5, 0.5), and [0.5,1]. An obvious phenomena was that there were fewer pairs of microbiota which remained weakly correlated in the Type 1 group than in the Type 2 group (Additional file 7: Figure S6).

**Fig. 7:**
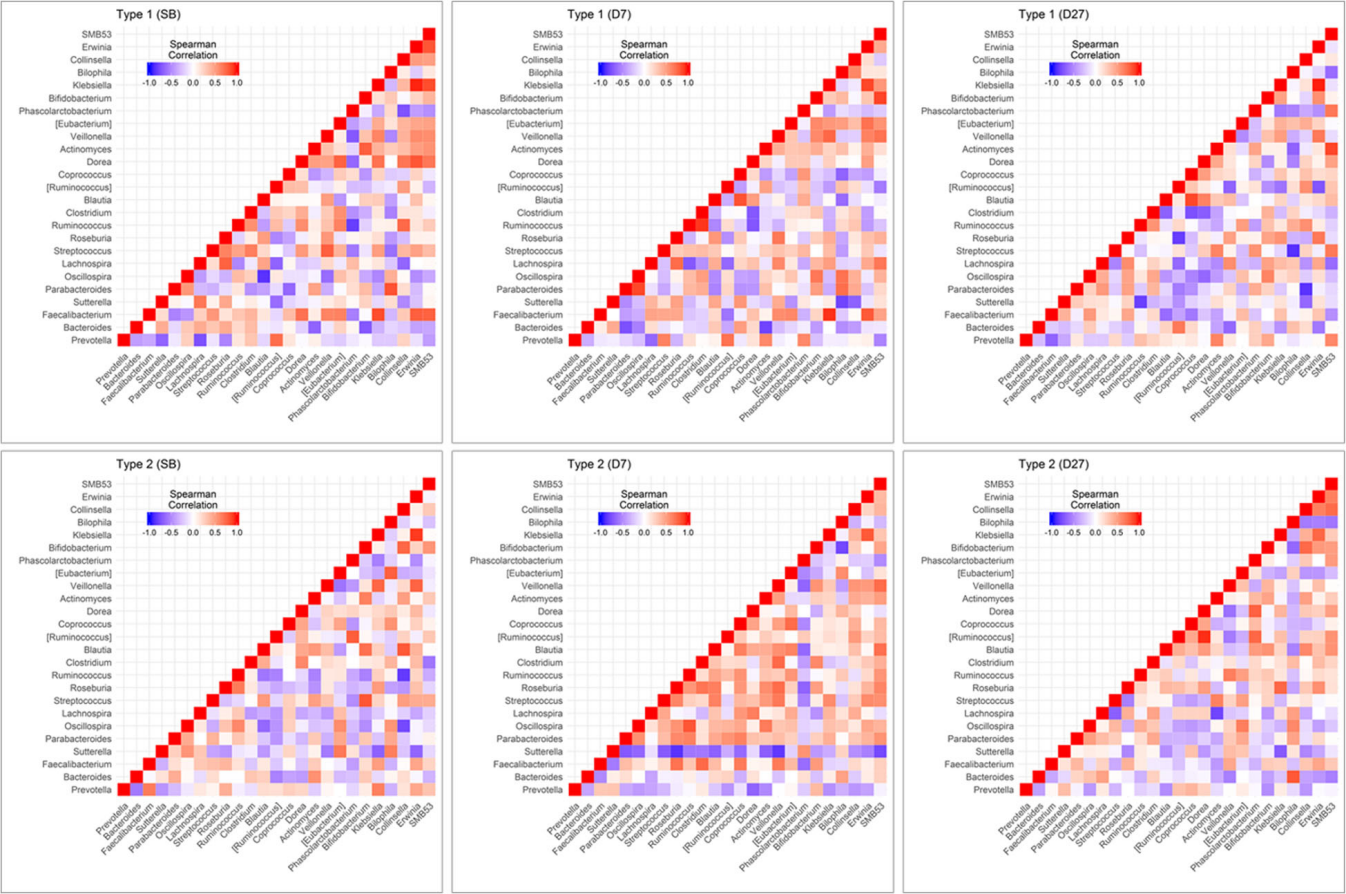
Six heat maps of correlation networks. Six heat maps of 25 genera which were detected in at least 90% of all 60 fecal samples. The pattern of theses microbial correlations within the Type 2 group is remarkably different at D7 compared with the other two collection time. Whereas these microbial correlations within the Type 1 group seemed not to change significantly

We also found that the correlation of 25 genera, which were detected in at least 90% of all 60 fecal samples, had different patterns. From six heat maps of the correlation networks, it is obvious that the pattern of these microbial correlations within the Type 2 group was remarkably different at D7 compared with the other two collection times, whereas these microbial correlations within the Type 1 group seemed not to change significantly (Fig. 7 and Additional file 8: Table S4-S9). It would appear that participants whose fecal type was the Type 2 were temporarily susceptible to bowel preparation.

### Variation of predictive functional modules in microbial community between the two types

Functional potential profiling of microbial communities was evaluated using PICRUSt (phylogenetic investigation of communities by reconstruction of unobserved states) via the KEGG database. At level 3 of the KEGG pathway database, 220 functional modules presented in the SB, D7, and D28 samples. In the SB fecal sample, 26 functional modules were significantly different between the Type 1 group and the Type 2 group (*P* < 0.05), of which mineral absorption and arachidonic acid metabolism differed remarkably (*P* = 1.19 × 10^− 5^ and *P* = 8.34 × 10^− 5^, respectively). (Additional file 9: Table S10).

**Fig. 8:**
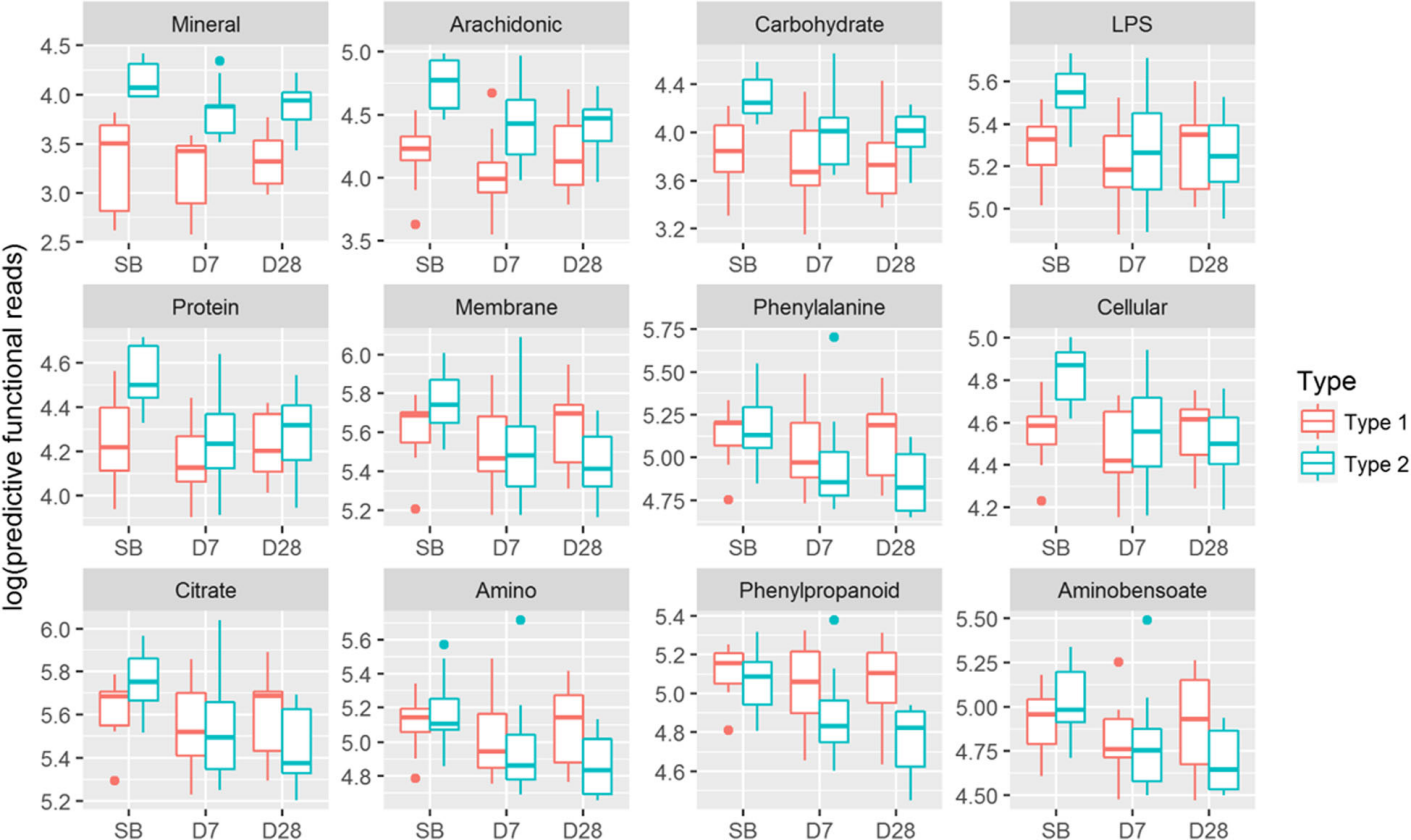
Boxplots of 12 predictive functional modules. The 12 predictive functional modules differed significantly either between the Type 1 and Type 2 groups, or over the three collection times (*P <* 0.05). The full module names are abbreviated as follows (1) Mineral = Mineral absorption; (2) Arachidonic = Arachidonic acid metabolism; (3) Carbohydrate = Carbohydrate digestion and absorption; (4) LPS = Lipopolysaccharide biosynthesis; (5) Protein = Protein digestion and absorption; (6) Membrane = Membrane and intracellular structural molecules; (7) Phenylalanine = Phenylalanine metabolism; (8) Cellular = Cellular antigens; (9) Citrate = Citrate cycle and TCA cycle; (10) Amino = Amino acid metabolism; (11) Phenylpropanoid = Phenylpropanoid biosynthesis; (12) Aminobensoate = Aminobensoate degradation

**Fig. 9:**
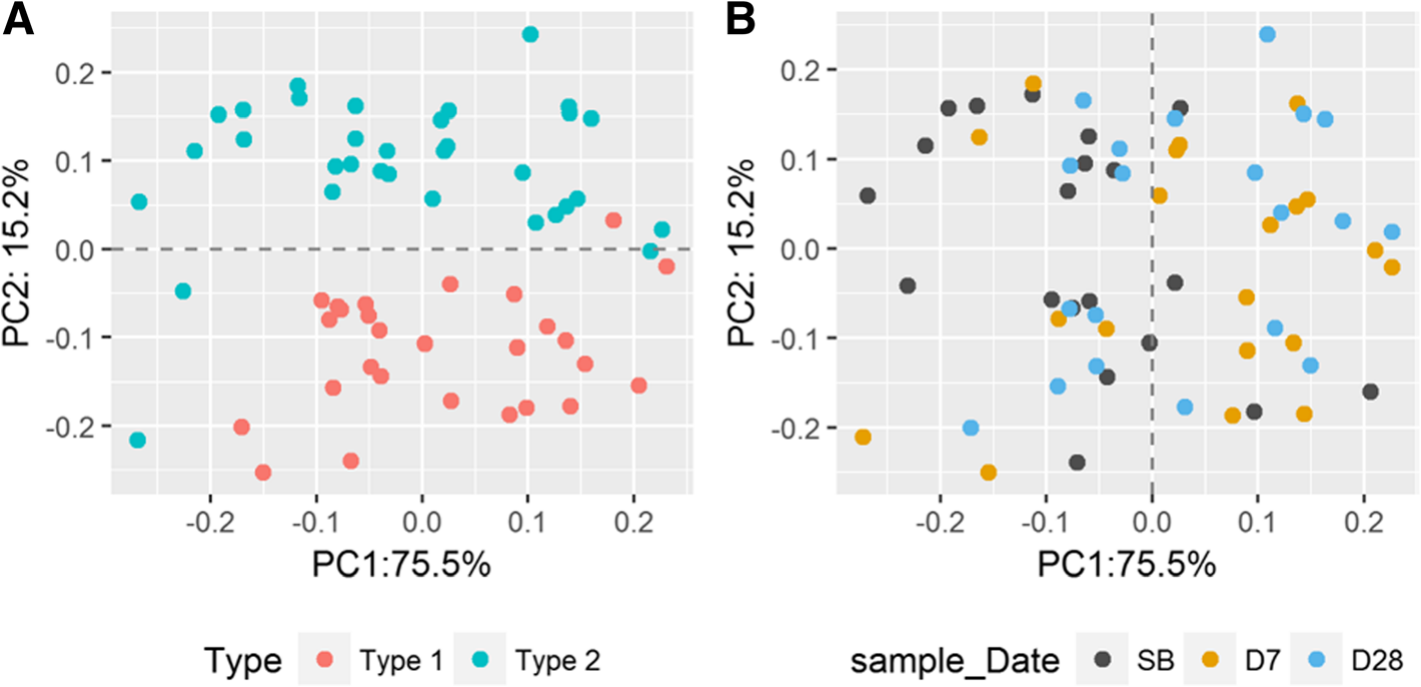
Principal component analysis of 12 predictive functional modules using PICRUSt in level 3 KEGG database. Each sample is represented by a colored point. (**a**) Red means type 1 samples, and blue means type 2 samples. The 60 fecal samples are mainly separated into two clusters. (**b**) Three colors denote the three different sample collection times. Gray means prior to bowel preparation, denoted as SB; orange means 7 days after colonoscopy, denoted as D7; and blue means 28 days after colonoscopy, denoted as D28. The SB and D7 fecal samples are roughly separated into two parts: the first part consists of 80% of the SB samples; the second part consists of 70% of the D7 samples. As for the D28 samples, 55% of them are closer to the predominantly SB part and 45% are in the predominantly D7 part

In the Type 1 group, of 220 functional modules, only one module (Bacterial invasion of epithelia cells) changed significantly from SB to D28 (Additional file 10: Table S11). Whereas, in the Type 2 group, 174 functional modules differed significantly between SB and D7 (*P* < 0.05); and 187 between SB and D28 (*P* < 0.05), of which lipopolysaccharide biosynthesis changed remarkably (*P* = 0.00018, Additional file 11: Table S12). Figure 8 depicts 12 functional modules which differed significantly between the Type 1 and Type 2 groups for each of the three collection times. Using principal component analysis, the 60 fecal samples were mostly separated into two clusters corresponding to the type (Fig. 9A). We also found that the SB and D7 fecal samples were roughly separated into two parts: the first part consisted of 80% of the SB fecal samples; the second part consisted of 70% of the D7 fecal samples. As for the D28 fecal samples, 55% of them were closer to the predominantly SB part and 45% were in the predominantly D7 part (Fig. 9B).

### Association of each type with inflammation cytokine and blood tests

In our blood samples, the averages of these inflammation cytokine, including C-reactive protein (CRP), interleukin 6 (IL-6), and tumor necrosis factor alpha (TNF-alpha), were within normal range, but the distribution of the test results differed in the participants of the two types. At all three time points, the participants in the Type 2 group generally had higher median for CRP, IL-6, and TNF-alpha than ones in the Type 1 group (Additional file 12: Figure S7 and Additional file 13: Table S13). In addition, the variance of CRP between the Type 1 and Type 2 groups was statistically significant at each sample collection time (*P* < 0.05, Additional file 13: Table S13). For each type, by comparing individually CRP, IL-6 and TNF-alpha, there is no statistically significant over the three collection times.

We also found that the variances of liver functions (serum glutamic oxaloacetic transaminase, also known as SGOT; and serum glutamic pyruvic transaminase, also known as SGPT), and the variance of basophils between the two types was significantly different at D7 and at D28 (*P* < 0.05, Additional file 14: Table S14). When comparing each type individually over the three collection times, only the variance of albumin differed significantly in the Type 2 group. Whereas the variance of the other blood tests between the two types generally did not change remarkably (Additional file 14: Table S15). The descriptive statistics of the blood test results present in Additional file 15: Table S16.

**Fig. 10:**
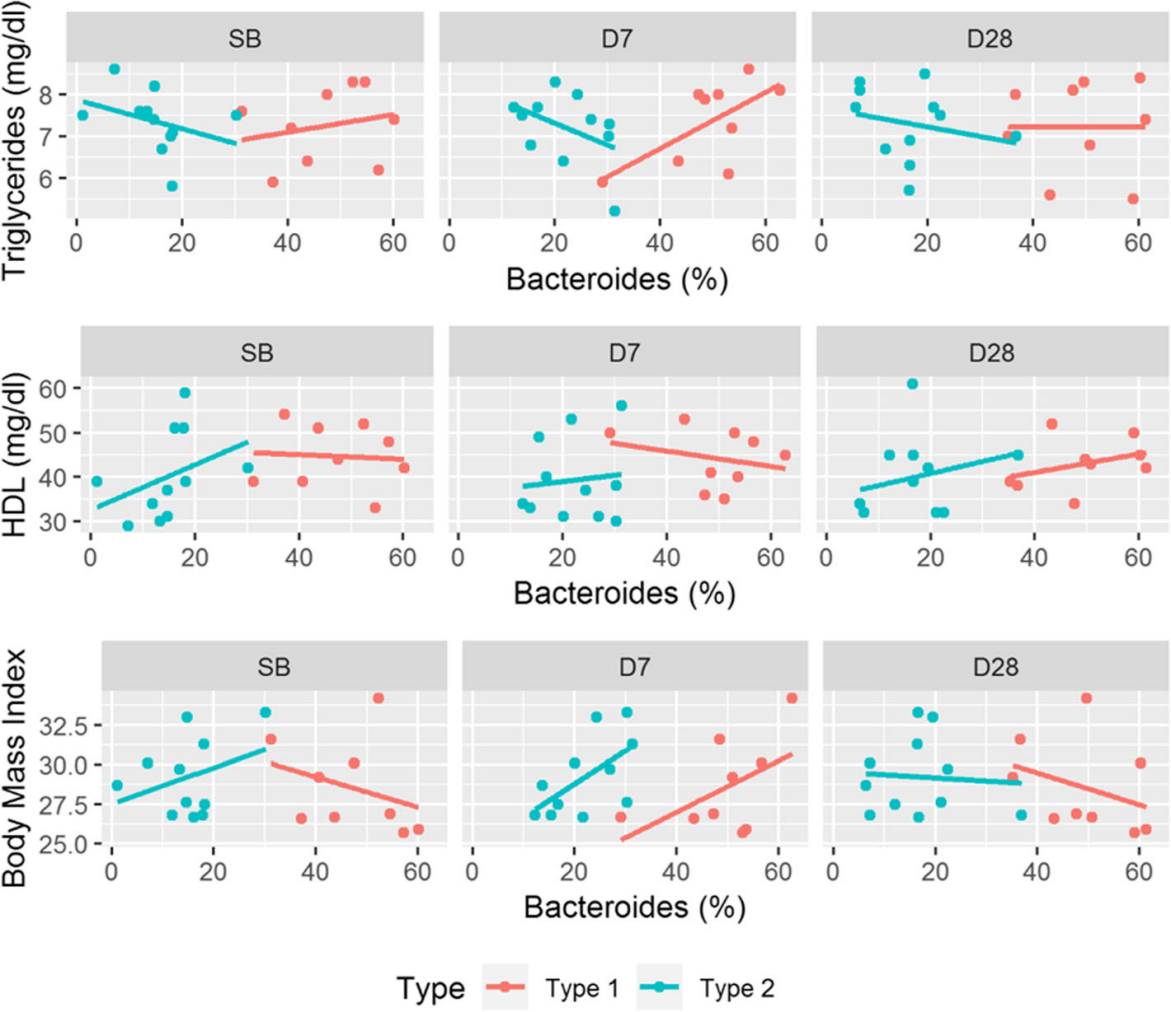
Scatter plots between *Bacteroides* abundance and Triglycerides, HDL, and Body Mass Index. HDL means high-density lipoproteins. Each subject is represented by a colored point. The correlations differed from fecal type at each sample collection time

Figure 10 depicts the correlations between *Bacteroides* abundance and each of TG, HDL, and BMI. There were three noteworthy phenomena. First, at each of the sample collection time, *Bacteroides* negatively correlated with TG in the Type 2 group, whereas *Bacteroides* positively correlated with TG in the Type 1 group. Furthermore, at SB, *Bacteroides* positively correlated with HDL in the Type 2 group (ρ = 0.66, *P* < 0.1) but very weakly negatively correlated with HDL in the Type 1 group (ρ = − 0.08) without statistical significance. Finally, the correlation between *Bacteroides* and BMI differed for each type at SB and D7. It was positive in the Type 2 group at SB and D7. In contrast, it was negative in the Type 1 group at SB but positive at D7.

## Discussion

The purpose of the study was to explore the gut microbiome change in overweight male adults. Our results demonstrated that the most dominant bacteria were hardly influenced by bowel preparation within individuals. However, microbial correlation networks within fecal types were somewhat different over the three sample collection times. In addition, some predictive functional models for microbial community and blood biochemical results within individuals differed between fecal types.

Clearing the bowel with a controlled diet and a laxative drink before colonoscopy may affect the normal gut microbiome for a short period of time. A study showed that the composition of gut microbiota in 10 middle-aged patients differed significantly after bowel preparation for at least a month [29]. Another case-control study proved that bowel preparation affected the diversity of the fecal and luminal microbiota for weeks [28]. Our results are not fully consistent with the previous findings. In our study, the most dominant bacteria abundances did not change much from prior to bowel preparation to 28 days after colonoscopy. The main reason may be that our participants were all male overweight adults with a healthy colon. In contrast, the subjects of the previous studies did not limit gender and colorectal health status.

Unlike the clustering methods of determining three enterotypes of the human gut microbiome [31, 38], in our study, we used *Prevotella* relative abundance divided by the sum of *Prevotella* and *Bacteroides* (P/(P + B)), for to the following reason. Our participants were overweight, and their fecal samples were dominated by either *Bacteroides* or *Prevotella*. According to the results of two recent studies, *Prevotella* and *Bacteroides* could be biomarkers of diet [39]; and enterotypes could be inferred simply by a *Prevotella*-to-*Bacteroides* ratio in persons with central obesity [32]. Thus, we modified a *Prevotella*-to-*Bacteroides* (P/B) ratio into P/(P + B) ratio, giving a range from 0 to 1, which allows us to directly compare the dominance of *Prevotella* or *Bacteriodes*.

*Bacteroides* and *Prevotella* belong to the phylum Bacteroidetes, which is the most stable component of gastrointestinal microbiota over time in healthy adults [40, 41]. Although our participants were overweight, they were, in general, healthy. Our result is consistent with the previous finding (Fig. 3, Fig. 6A). Another study also showed that the reduced abundance of the Bacteroidetes was linked with obesity [9, 10, 42]. In our study, over the three sample collection times, the correlation between Bacteroidetes and Firmicutes in the Type 1 group changed remarkably while remaining mostly unchanged in the Type 2 (Fig. 6C).

Early research reported that *Bacteroides* was more common in Western populations [43] and that *Prevotella* was more common in non-Western populations such as Papua New Guineans [44, 45]. Some researchers further pointed out the potential of reshape the metabolism of *Bacteroiedes* [46]. In our study, the Type 1 fecal samples were dominated by *Bacteroides*; and the Type 2 fecal samples were dominated by *Prevotella* with a noticeable presence of *Bacteroides.* Furthermore, the proportions of *Bacteroides*-dominant fecal samples and *Prevotella*-dominant fecal samples were similar. Additionally, the two predictive functional modules, “carbohydrate digestion and absorption” and “mineral absorption”, in the Type 2 group had higher levels than in the Type 1 group (Fig. 8). Because our participants were omnivorous and we lacked their diet records, it was a challenge to figure out what causes this phenomenon. However, in Taiwan, the staple diet is white rice [47]. It is rich in carbohydrate and contains minerals such as magnesium, phosphorus, manganese, selenium, iron, folic acid, and thiamine. Further comparing their occupations, we found that the participants in the Type 1 group were all sedentary workers, including programmers, researchers, and project managers. Whereas in the Type 2 group, almost half of the participants were non-office workers, including drivers, manual laborers, and salesmen (Additional file 16: Table S17). Thus, the participants in the Type 2 group might need to ingest more carbohydrate-associated food such as white rice than the participants in the Type 1 group due to the need for more calories for labor.

People who are either overweight or obese are at high risk of metabolic diseases [8]. *Akkermansia*, *Bulleidia*, and *SMB53* are reported to be associated with obesity. The only current known species within genus *Akkermansia* is *Akkermansia muciniphila*, which is a mucin-degrading bacterium [48]. Recent studies indicated that it is associated with body fat mass and glucose intolerance in mice, and that higher *A. muciniphila* seems to be a healthier metabolic status in overweight/obese humans [49]. As for the *Bulleidia*, it is more abundant in samples with type 2 diabetes (T2DM) and in the early stages of Prediabetes compared to Non-Diabetic samples [50]. In addition, resistant starch or extruded grain result in the enrichment of the relative abundance of *Bulleidia* [51–53]. The genus *SMB53* belongs to the family Clostridiaceae; and a mice model suggests it may be an important factor for the abnormal metabolism of T2DM [54].

In our study, both *Akkermansia* and *Bulleidia* differed significantly between the Type 1 and Type 2 fecal samples at SB (*P* < 0.05) but *SMB53* did not (Additional file 17: Table S18). Furthermore, the proportions of these genera in each type fecal group were different. At SB fecal samples, *Akkermansia* appeared in 22% of the Type 1 and 73% of the Type 2; *Bulleidia* appeared in 89% of the Type 1 and 55% of the Type 2; and *SMB53* appeared in almost all fecal samples. Additionally, the proportions of *Akkermansia* and *SMB53* in each type were similar over the three sample collection times, but that of *Bulleidia* in the Type 2 group declined remarkably at D7. It seemed that *Akkermansia* is more likely to appear in the Type 2 fecal samples; and that *Bulleidia* is more likely to appear in the Type 1 fecal samples. Therefore, it is noted that *Akkermansia*, *Bulleidia*, and *SMB53* play a role in overweight people who are dominated either by *Bacteroides* or *Prevotella*. However, the influence and interaction between these three genera is unclear and requires further investigation.

Recent research showed that some gut bacteria caused substantial variations in triglycerides (TG), high-density lipoproteins (HDL), and BMI [55]. Consistent with this, we also found that *Bacteroides*, in our study, negatively correlated with TG; positively correlated with HDL; and very weakly correlated with BMI. However, when we separated the participants into two groups based on the *Prevotella*-ratio, *Bacteroides* correlated more strongly with each of TG, HDL, and BMI in the Type 2 group than in the Type 1 group at SB and D7(Fig. 10).

Although we focus here on the changes and variations of fecal microbiome in overweight male adults, these findings do not determine the contributions of each genus to the nutrition and health of the host. This study has some limitations. First, due to the number of samples, the findings of this study are restricted to the comparison of two groups corresponding to *Prevotella*-ratios within individuals. It could be coincident that the range of *Prevotella*-ratios was either lower than 0.1 or greater than 0.4. A larger sample size may yield *Prevotella*-ratios between these two values, or confirm the existence of this split. Second, details of dietary habits and lifestyles were not explored. Our participants were only asked if they were vegetarian or omnivore, and their response was recorded by medical doctors each time participants came back to the hospital during the study period. Further study should include a detailed dietary questionnaire. Then, at least, we could figure out the major differences in meal content between the Type 1 and Type 2 participants. Third, all of our participants were defined as overweight whose BMI were greater than or equal to 25 kg/m^2^. However, obese individuals, whose BMI are greater than or equal to 30 kg/m^2^, have an excess accumulation of fat, while overweight individuals may or may not. Thus, it might be better to study overweight and obese samples separately. Finally, 16S rRNA gene amplicon sequencing generates the relative abundance of taxa or genes within microbial community. It does not provide absolute abundance information. In addition, biases will occur from the sample processing pipeline [56]. For further study, we should assess the effects of biases for our particular choices of protocols.

## Conclusions

Depending upon the fecal type, the microbial diversity and the predictive functional modules of microbial community differed significantly after bowel preparation. In addition, blood biochemical markers presented somewhat associated with fecal type. Although it is still unclear whether the microbiota composition of feces can accurately reflect the real state of the microbiota composition in the intestine, the gut microbiota by fecal samples might provide clues for health status and might provide to promote health through personalized clinical treatment.

## Additional files

Additional file 1: **Table S1.** Exclusion criteria. (PDF 76 kb)

Additional file 2: **Table S2.** Percentage of fecal samples and mean relative abundances of the 131 genera. (XLSX 27 kb)

Additional file 3: **Figure S1.** The differences of microbial diversity between the type 1 and 2 groups. (PDF 331 kb)

Additional file 4: **Table S3.** Descriptive statistics of richness and Shannon diversity index. (PDF 184 kb)

Additional file 5: **Figure S2.** Principal coordinate analysis (PCoA) based on the UniFrac distance values. (PDF 414 kb)

Additional file 6: **Figsure S3-S5.** Correlation networks for 61 genera at three sample collection times. (PDF 2498 kb)

Additional file 7: **Figure S6**. Percentage of the number of correlation change between SB and S7; SB and S28. (PDF 232 kb)

Additional file 8: **Tables S4-S9**. Significant correlations for each type group at each sample collection time. (XLSX 27 kb)

Additional file 9: **Table S10.** Means of 26 predictive functional modules between the Type 1 and Type 2 fecal samples. (PDF 209 kb)

Additional file 10: **Table S11.** The *P*-values of the predictive functional modules of the Type 1 fecal microbial community. (XLSX 23 kb)

Additional file 11: **Table S12.** The P-values of the predictive functional modules of the Type 2 Fecal community. (XLSX 23 kb)

Additional file 12: **Figure S7.** Box plots of three inflammation cytokines. (PDF 377 kb)

Additional file 13: **Table S13.** Descriptive statistics of blood test. (XLSX 13 kb)

Additional file 14: **Tables S14 and S15.** Table S14: P-values between the Type 1 and Type 2 groups; **Table S15**: P-values between between any two collection times for each type Table. (PDF 430 kb)

Additional file 15: **Table S16.** Descriptive statistics of blood test. (XLSX 17 kb)

Additional file 16: **Table S17.** Participant’s lifestyle. (PDF 89 kb)

Additional file 17: **Table S18.** Percentage of fecal samples and mean relative abundance of *SMB53*, *Bulleidia*, and *Akkermansia*. (PDF 176 kb)

## Abbreviations

16S rRNA: 16S ribosomal RNA
ALT: Serum alanine aminotransferase
AST: Aspartate aminotransferase
BMI: Body mass index
CDI: *Clostridium difficile* infections
CRP: C-reactive protein
FMT: Fecal material transplantation
HDL: High-density lipoprotein cholesterol
IDB: Inflammatory bowel disease
IL-6: Interleukin 6
OUT: Operational taxonomic units
PICRUSt: Phylogenetic investigation of communities by reconstruction of unobserved states
TG: Triglycerides
TNF-alpha: Tumor necrosis factor alpha
