# Supplementary material for: Gut microbiome changes in overweight male adults following bowel preparation
Source: BMC Genomics. 2018 Dec 31;19(Suppl 10):904. doi: 10.1186/s12864-018-5285-6 (PMC6311932; doi:10.1186/s12864-018-5285-6)
Supplement: Supplementary file 1 — Table S1. Exclusion criteria. (PDF 76 kb) [file 12864_2018_5285_MOESM1_ESM.pdf]

Table S1. Exclusion criteria

I. Medication within one month prior to informed consent<br>1 Antibiotic<br>2. Probiotic, prebiotic, symbiotic, or any food and drink containing probiotics<br>3. Anti-inflammatory drugs<br>4. Weight-loss medication<br>5. Systemic steroid<br>6. Medication for endocrine disorder<br>7. Alcohol or drug abuse
II. Medical history of<br>1. Myocardial infarction or stroke within 3 months prior to informed consent.<br>2. Liver disease<br>3. Chronic kidney disease with stage higher than 3B<br>4. Inflammatory gastrointestinal disorders such as Crohn’s disease or colitis.<br>5. Cancer<br>6. Gastrointestinal tract surgeries
III. Take an investigational drug within 30 days prior to informed consent
IV. Any other clinical condition that would jeopardize subjects’ safety while participating in this clinical trial
