# Supplementary material for: Gut microbiome changes in overweight male adults following bowel preparation
Source: BMC Genomics. 2018 Dec 31;19(Suppl 10):904. doi: 10.1186/s12864-018-5285-6 (PMC6311932; doi:10.1186/s12864-018-5285-6)
Supplement: Supplementary file 3 — Figure S1. The differences of microbial diversity between the type 1 and 2 groups. (PDF 331 kb) [file 12864_2018_5285_MOESM3_ESM.pdf]

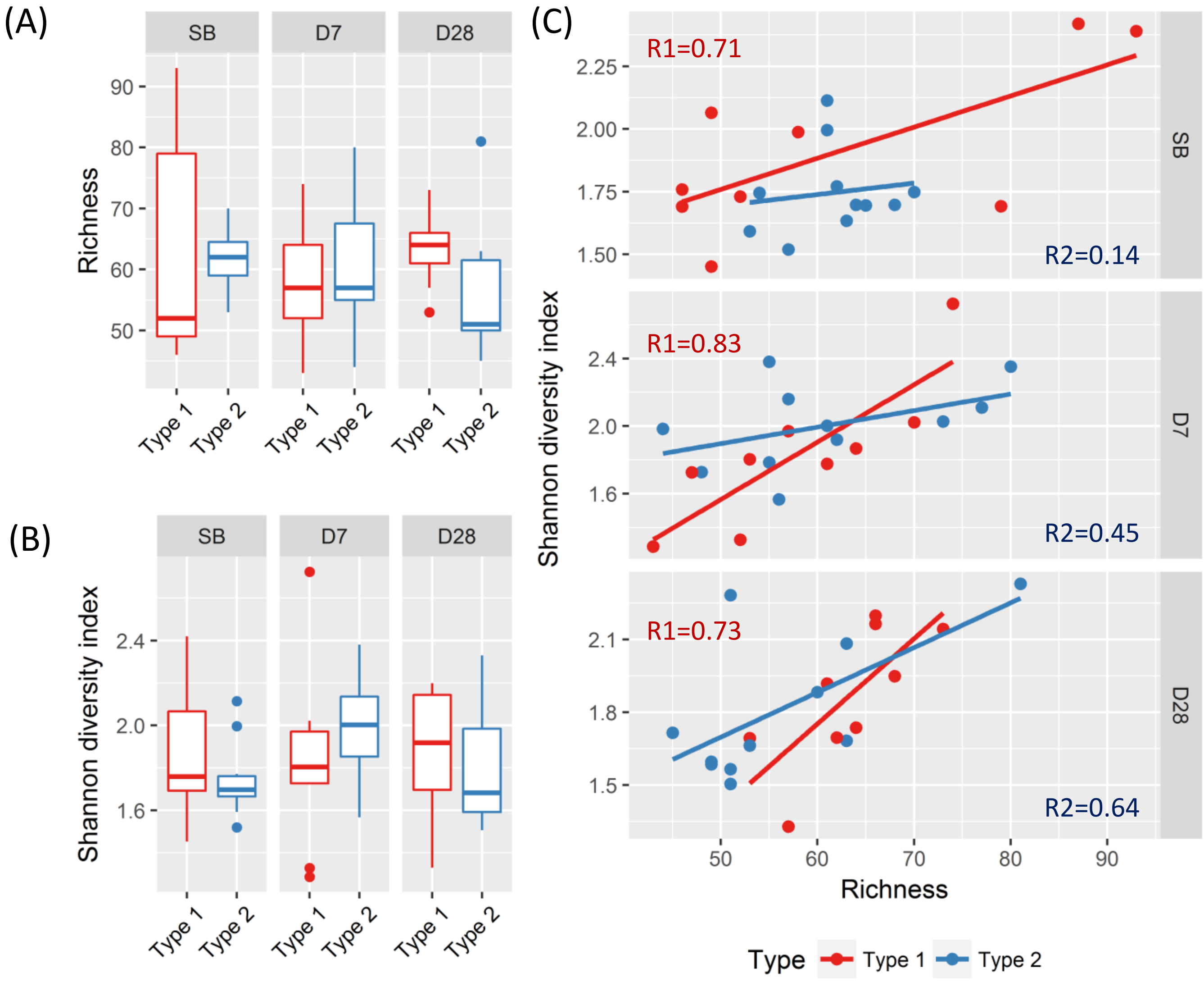

Figure S1. The differences of microbial diversity between the type 1 and 2 groups. (A) Boxplot of richness. In D28 samples, Type 1 and Type 2 differ significantly (p-value: 0.025). (B) Boxplot of Shannon diversity index. There is no statistical difference between the two types at each of the three collection times. (C) Correlation plots of richness and Shannon diversity index for each type at each collection time. The correlation coefficient between richness and Shannon diversity index of each type became closer from SB to D28.
