# Supplementary material for: Gut microbiome changes in overweight male adults following bowel preparation
Source: BMC Genomics. 2018 Dec 31;19(Suppl 10):904. doi: 10.1186/s12864-018-5285-6 (PMC6311932; doi:10.1186/s12864-018-5285-6)
Supplement: Supplementary file 4 — Table S3. Descriptive statistics of richness and Shannon diversity index. (PDF 184 kb) [file 12864_2018_5285_MOESM4_ESM.pdf]

Table S3. Descriptive statistics of richess and Shannon diversity index

	SB		D7		D28	
	Type 1	Type 2	Type 1	Type 2	Type 1	Type 2
Richness (Min–Max)	46–93	53–70	43–74	44–80	53–73	45–81
mean±SD	62 ± 18.8	62 ± 5.3	58 ± 10.3	61 ± 11.5	63 ± 7.0	56 ± 10.2
median	52	62	57	57	64	51
P-value	0.323		0.594		*0.025	

	SB		D7		D28	
	Type 1	Type 2	Type 1	Type 2	Type 1	Type 2
Shannon diversity index	1.45–2.42	1.52–2.11	1.33–2.72	1.57–2.38	1.33–2.20	1.51–2.33
mean±SD	1.91 ± 0.33	1.75 ± 0.17	1.83 ± 0.42	2.00 ± 0.25	1.87 ± 0.29	1.81 ± 0.29
median	1.76	1.70	1.80	2.00	1.91	1.68
P-value	0.412		0.152		0.37	

	**P-values for richness**		
	SB vs. D7	D7 vs. D28	SB vs. D28
Type 1	1	0.215	0.331
Type 2	0.553	0.357	*0.032

	**P-values for Shannon index**		
	SB vs. D7	D7 vs. D28	SB vs. D28
Type 1	0.863	0.73	1
Type 2	*0.016	0.088	0.847

*Abbreviation: SD, standard deviation

*P-values are calculated by the Wilcoxon rank-sum test
