# Supplementary material for: Gut microbiome changes in overweight male adults following bowel preparation
Source: BMC Genomics. 2018 Dec 31;19(Suppl 10):904. doi: 10.1186/s12864-018-5285-6 (PMC6311932; doi:10.1186/s12864-018-5285-6)
Supplement: Supplementary file 5 — Figure S2. Principal coordinate analysis (PCoA) based on the UniFrac distance values. (PDF 414 kb) [file 12864_2018_5285_MOESM5_ESM.pdf]

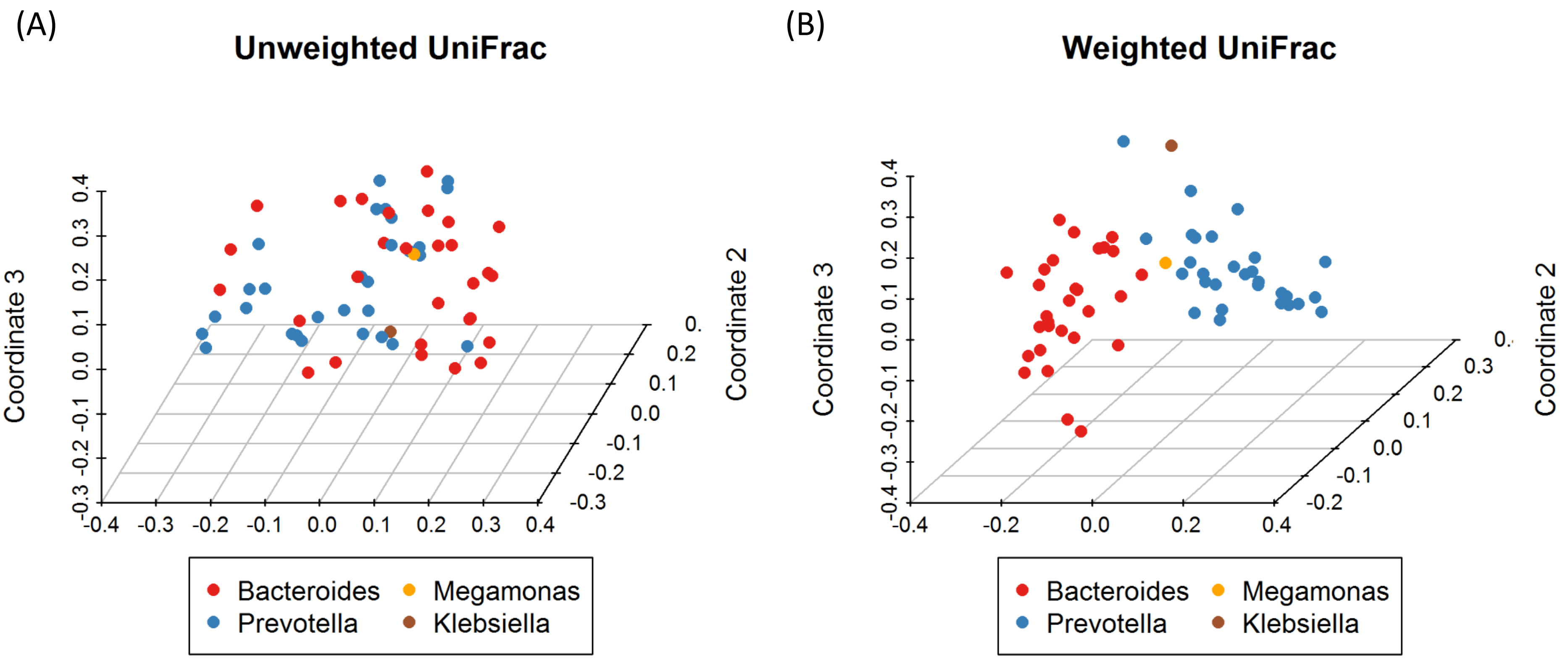

Figure S2. Principal coordinate analysis (PCoA) based on the UniFrac distance values. Each point represents one fecal sample. Each color means the most abundant genus within individuals. (A) Unweighted UniFrac Distance. (B) Weighted UniFrac Distance. From (B), 58 fecal samples were separated into two clusters by their predominant genus *Bacteroides* and *Prevotella*, of which only two samples, at baseline and 7-days after colonoscopy examination, were dominated by *Megomonas* and *Klebsiella*, while at the same their microbial diversity were similar with *Prevotella*-dominant samples .
