# Supplementary material for: Gut microbiome changes in overweight male adults following bowel preparation
Source: BMC Genomics. 2018 Dec 31;19(Suppl 10):904. doi: 10.1186/s12864-018-5285-6 (PMC6311932; doi:10.1186/s12864-018-5285-6)
Supplement: Supplementary file 6 — Figsure S3-S5. Correlation networks for 61 genera at three sample collection times. (PDF 2498 kb) [file 12864_2018_5285_MOESM6_ESM.pdf]

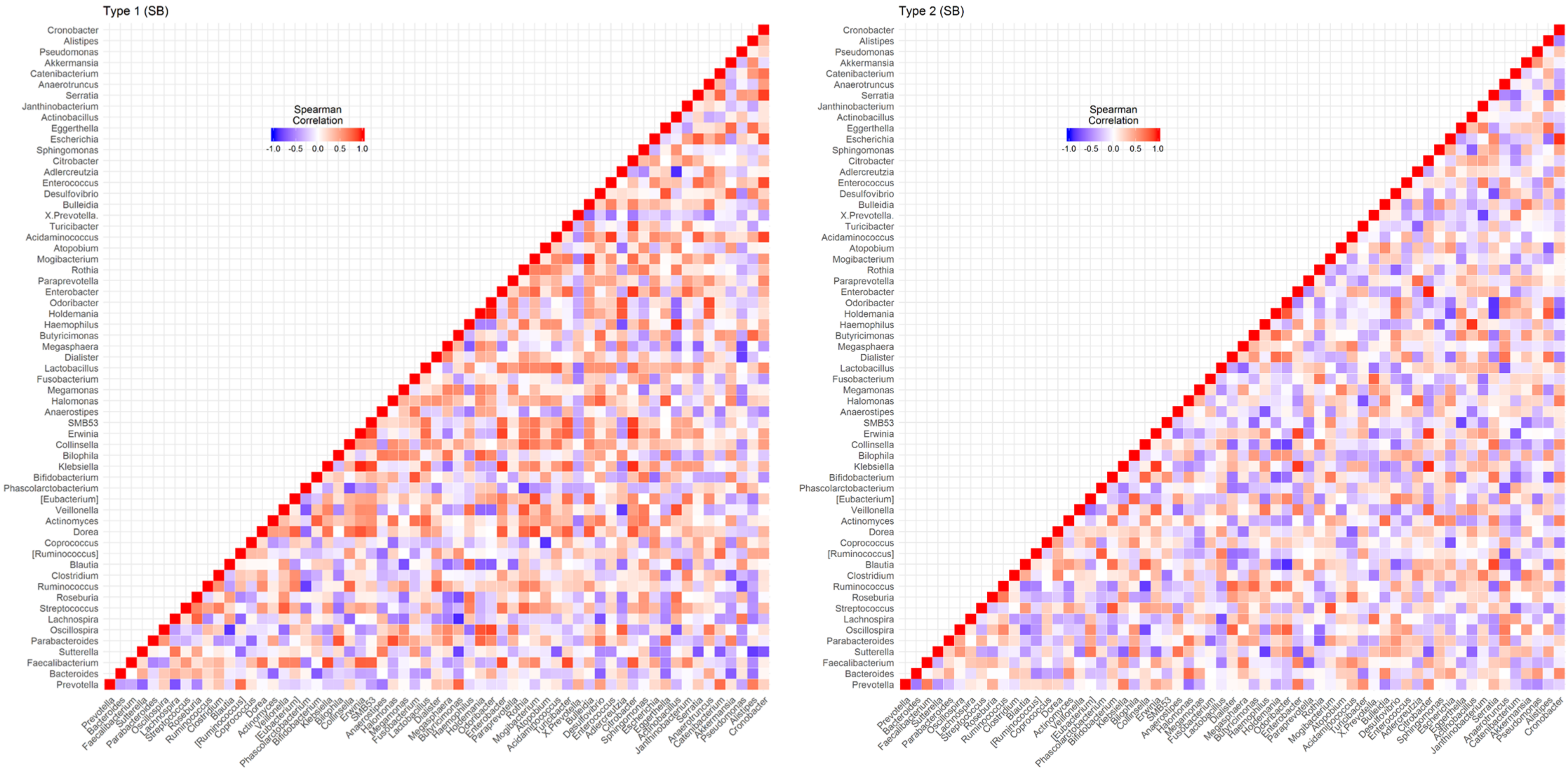

Figure S3. Correlation networks for 61 genera at baseline. SB means the sample collection time prior to bowel prep, which is baseline. The strength of correlation was evaluated using Spearman correlation coefficients. Red color means positive correlation, and blue color means negative correlation. Bacterial names are arranged in descending order according to the percentage of sample present. The correlation of 61 genera had different patterns between the Type 1 group and the Type 2 group.

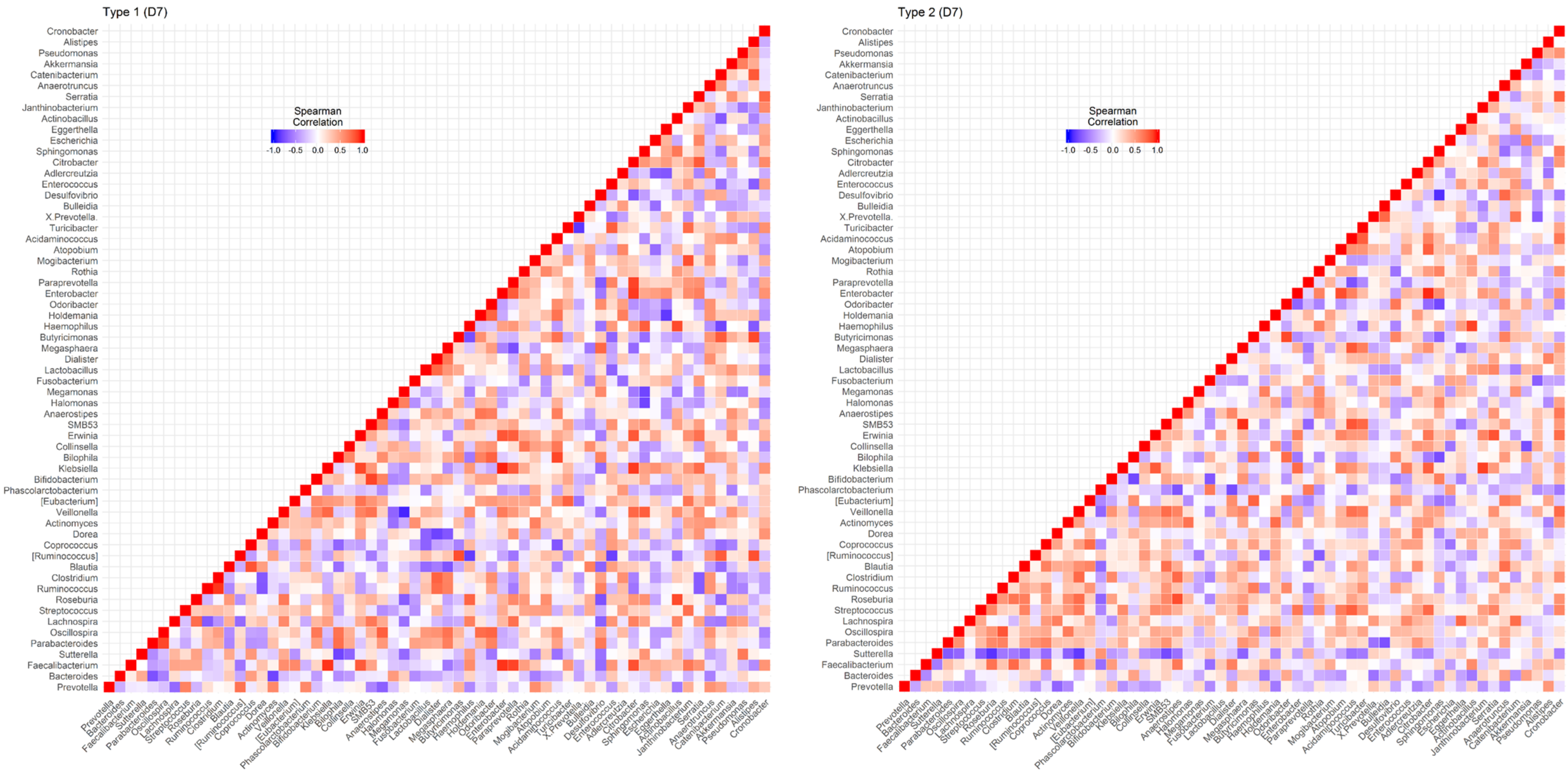

Figure S4. Correlation networks for 61 genera 7 days after colonoscopy. D7 means the sample collection 7 days after colonoscopy. The strength of correlation was evaluated using Spearman correlation coefficient. Red color means positive correlation, and blue color means negative correlation. Bacterial names are arranged in descending order according to the percentage of sample present.

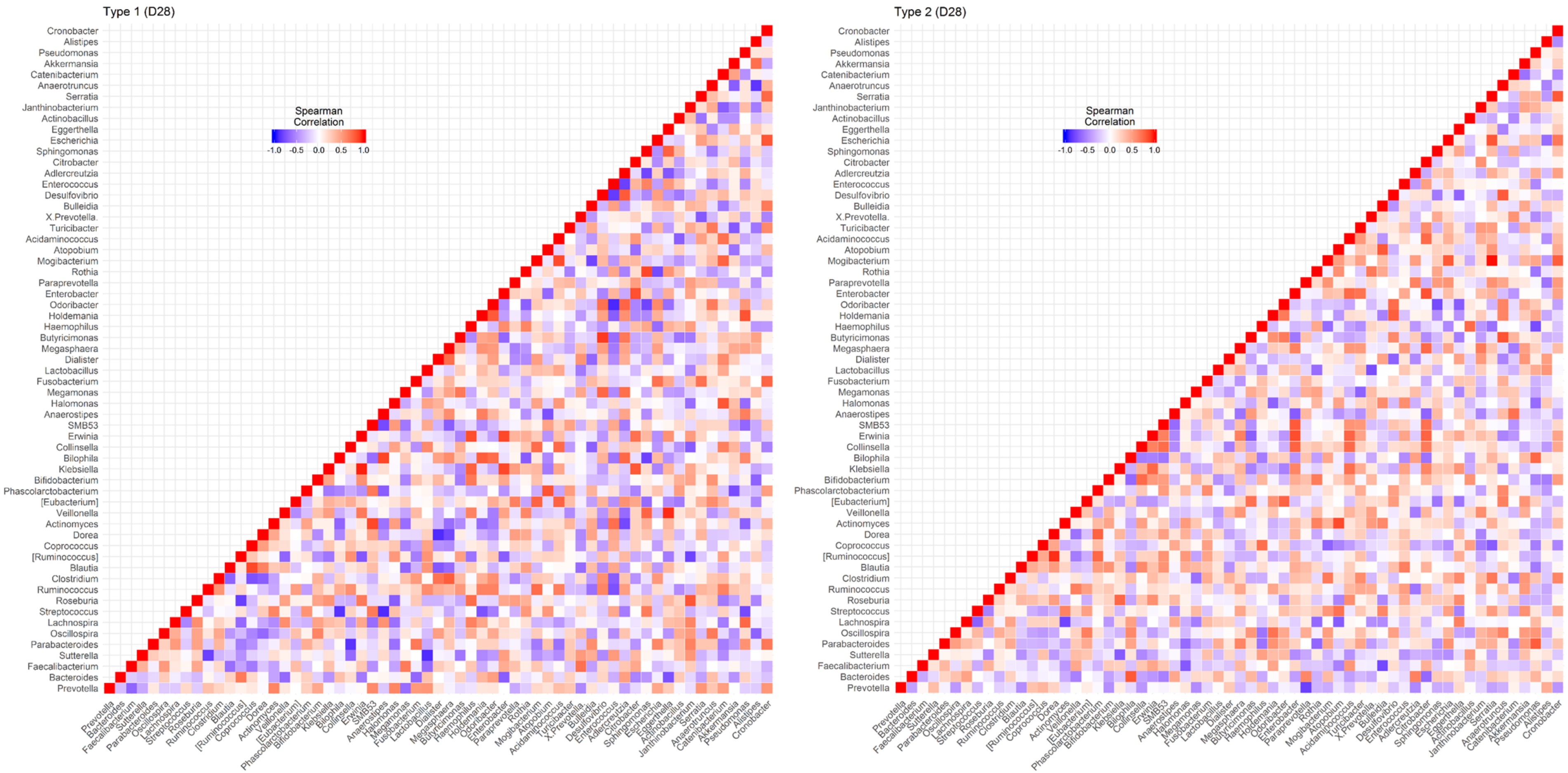

Figure S5. Correlation networks for 61 genera 28 days after colonoscopy. D28 means the sample collection 28 days after colonoscopy. The strength of correlation was evaluated using Spearman correlation coefficient. Red color means positive correlation, and blue color means negative correlation. Bacterial names are arranged in descending order according to the percentage of sample present.
