# Supplementary material for: Gut microbiome changes in overweight male adults following bowel preparation
Source: BMC Genomics. 2018 Dec 31;19(Suppl 10):904. doi: 10.1186/s12864-018-5285-6 (PMC6311932; doi:10.1186/s12864-018-5285-6)
Supplement: Supplementary file 7 — Figure S6. Percentage of the number of correlation change between SB and S7; SB and S28. (PDF 232 kb) [file 12864_2018_5285_MOESM7_ESM.pdf]

Type 1	D7	D7	D7
SB	1.37%	4.86%	0.05%
SB	5.25%	65.52%	7.38%
SB	0.27%	10.60%	4.70%

Type 1	D28	D28	D28
SB	1.04%	5.08%	0.16%
SB	8.14%	63.83%	6.18%
SB	0.49%	12.24%	2.84%

Type 2	D7	D7	D7
SB	0.71%	6.01%	0.11%
SB	4.54%	72.02%	8.58%
SB	0.11%	6.12%	1.80%

Type 2	D28	D28	D28
SB	0.77%	5.96%	0.11%
SB	4.64%	74.92%	5.57%
SB	0.16%	5.52%	2.35%

Spearman Correlation	
	[−1, −0.5]
	(−0.5, 0.5)
	[0.5, 1]

Figure S6. Percentage of the numbers of correlation change between SB and S7; SB and S28. 61 genera were detected in at least 50% of the fecal subjects at a particular collection time. The strength of correlation was evaluated using Spearman's correlation coefficient. The 1830 correlation coefficients are separated into three subintervals [−1, −0.5], (−0.5, 0.5), and [0.5, 1]. Blue color represents the first subinterval, orange the second, and red the third. Each number in each cell of the four tables means the percentage of the number of the pair of genera whose correlations changed from each subinterval to any one of the three subintervals. There are fewer pairs of microbiota which remained weakly correlated in the Type 1 group than in the Type 2 group.
