# Supplementary material for: Gut microbiome changes in overweight male adults following bowel preparation
Source: BMC Genomics. 2018 Dec 31;19(Suppl 10):904. doi: 10.1186/s12864-018-5285-6 (PMC6311932; doi:10.1186/s12864-018-5285-6)
Supplement: Supplementary file 9 — Table S10. Means of 26 predictive functional modules between the Type 1 and Type 2 fecal samples. (PDF 209 kb) [file 12864_2018_5285_MOESM9_ESM.pdf]

Table S10. Means of 26 predictive functional modules between the Type 1 and Type 2 fecal samples

		SB				D7				D28			
NO	KEGG (Level 3)	Type 1	Type 2	P-value	adj.P	Type 1	Type 2	P-value	adj.P	Type 1	Type 2	P-value	adj.P
1	Mineral absorption	7.5824	9.5733	0.00001	0.00262	7.3507	8.8004	0.00005	0.01048	7.6467	8.9129	0.00080	0.17552
2	Arachidonic acid metabolism	9.6366	10.9148	0.00008	0.01834	9.2958	10.1869	0.01247	1.00000	9.6609	10.1617	0.15194	1.00000
3	Carbohydrate digestion and absorption	8.8169	9.9184	0.00080	0.17552	8.6162	9.1864	0.11194	1.00000	8.7352	9.1548	0.15194	1.00000
4	Cellular antigens	10.4850	11.0857	0.00116	0.25411	10.2878	10.5010	0.37022	1.00000	10.4765	10.3843	0.60268	1.00000
5	Lipopolysaccharide biosynthesis	12.1966	12.7818	0.00164	0.36151	12.0069	12.1849	0.65563	1.00000	12.1769	12.0935	0.82376	1.00000
6	Protein digestion and absorption	9.7715	10.4634	0.00424	0.93260	9.6069	9.8311	0.33116	1.00000	9.7208	9.8446	0.45610	1.00000
7	Zeatin biosynthesis	10.4020	10.8721	0.00969	1.00000	10.2420	10.3382	0.82376	1.00000	10.3430	10.2558	0.60268	1.00000
8	Prenyltransferases	12.1615	12.5501	0.01590	1.00000	11.9812	12.0346	1.00000	1.00000	12.1031	11.9262	0.29472	1.00000
9	Lipopolysaccharide biosynthesis proteins	12.5620	13.0145	0.02004	1.00000	12.3367	12.4271	0.82376	1.00000	12.5466	12.3007	0.33116	1.00000
10	Homologous recombination	13.1951	13.5237	0.02505	1.00000	13.0319	13.0411	0.94084	1.00000	13.1533	12.9035	0.13080	1.00000
11	DNA replication	12.8505	13.1634	0.02505	1.00000	12.6800	12.6845	1.00000	1.00000	12.8034	12.5510	0.13080	1.00000
12	Riboflavin metabolism	11.9212	12.3582	0.02505	1.00000	11.7366	11.8017	0.94084	1.00000	11.9074	11.6814	0.41190	1.00000
13	Ribosome	14.0880	14.4502	0.03103	1.00000	13.9222	13.9641	0.88198	1.00000	14.0350	13.8365	0.20137	1.00000
14	Pyrimidine metabolism	13.8581	14.2137	0.03103	1.00000	13.6903	13.7208	1.00000	1.00000	13.8180	13.5869	0.15194	1.00000
15	DNA replication proteins	13.4728	13.7960	0.03103	1.00000	13.3030	13.3134	0.82376	1.00000	13.4322	13.1762	0.11194	1.00000
16	One carbon pool by folate	12.8298	13.2123	0.03103	1.00000	12.6609	12.7064	1.00000	1.00000	12.7775	12.5846	0.22986	1.00000
17	Translation factors	12.6340	12.9798	0.03103	1.00000	12.4650	12.4909	1.00000	1.00000	12.5914	12.3576	0.17541	1.00000
18	Peptidoglycan biosynthesis	13.0345	13.4020	0.03813	1.00000	12.8508	12.8937	1.00000	1.00000	12.9969	12.7704	0.20137	1.00000
19	Mismatch repair	13.0472	13.3662	0.03813	1.00000	12.8852	12.8786	0.88198	1.00000	13.0073	12.7506	0.13080	1.00000
20	Folate biosynthesis	12.4129	12.7539	0.03813	1.00000	12.2037	12.2141	0.94084	1.00000	12.3716	12.0893	0.15194	1.00000
21	Epithelial cell signaling in Helicobacter pylori infection	10.8626	11.2185	0.03813	1.00000	10.6902	10.7038	0.76643	1.00000	10.8227	10.5532	0.13080	1.00000
22	Vibrio cholerae pathogenic cycle	10.7168	11.1400	0.03813	1.00000	10.5187	10.5643	0.94084	1.00000	10.7641	10.4541	0.22986	1.00000
23	DNA repair and recombination proteins	14.2997	14.6166	0.04645	1.00000	14.1271	14.1304	0.82376	1.00000	14.2666	13.9882	0.11194	1.00000
24	Chromosome	13.7265	14.0649	0.04645	1.00000	13.5617	13.5610	0.76643	1.00000	13.7091	13.4242	0.09518	1.00000
25	Terpenoid backbone biosynthesis	12.7126	13.0537	0.04645	1.00000	12.5369	12.5596	1.00000	1.00000	12.6520	12.4303	0.17541	1.00000
26	Secondary bile acid biosynthesis	10.0014	9.5554	0.04645	1.00000	9.8936	9.2731	0.00164	0.36151	9.9726	9.0514	0.00080	0.17552

* The means are caluated by the 10-based logtransform of predictive functional reads
* The p-values are calculated by the Wilcoxon rank-sum test.
*ad.p: adjusted p-value using Bonferroni correction
