# Supplementary material for: Gut microbiome changes in overweight male adults following bowel preparation
Source: BMC Genomics. 2018 Dec 31;19(Suppl 10):904. doi: 10.1186/s12864-018-5285-6 (PMC6311932; doi:10.1186/s12864-018-5285-6)
Supplement: Supplementary file 12 — Figure S7. Box plots of three inflammation cytokines. (PDF 377 kb) [file 12864_2018_5285_MOESM12_ESM.pdf]

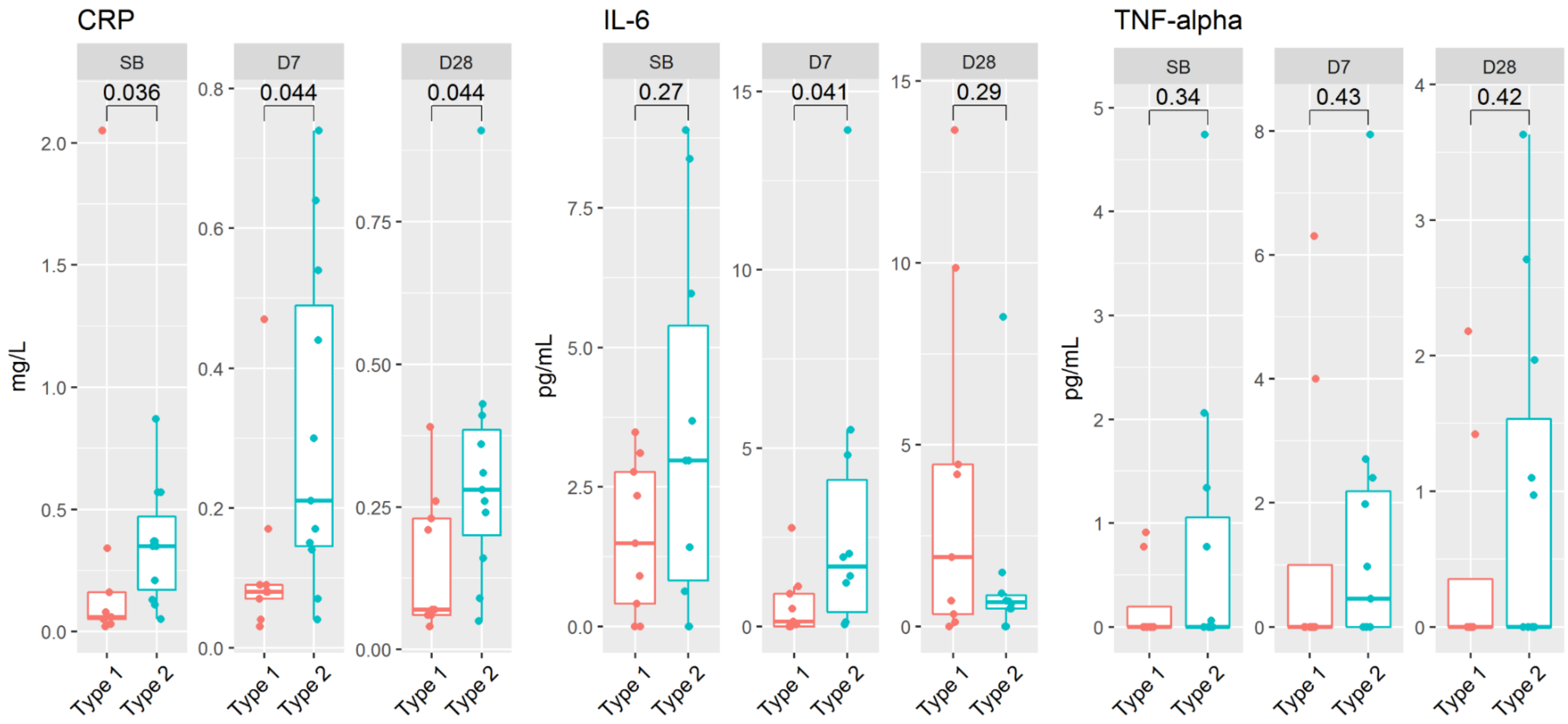

Figure S7. Box plots of three inflammation cytokines. At all three time points, the Type 2 group generally had higher median for CRP, IL-6, and TNF-alpha than the Type 1 group. For each type, by comparing individually CRP, IL-6 and TNF-alpha, there is no statistically significant over the three collection times.
