# Supplementary material for: Gut microbiome changes in overweight male adults following bowel preparation
Source: BMC Genomics. 2018 Dec 31;19(Suppl 10):904. doi: 10.1186/s12864-018-5285-6 (PMC6311932; doi:10.1186/s12864-018-5285-6)
Supplement: Supplementary file 14 — Tables S14 and S15. Table S14: P-values between the Type 1 and Type 2 groups; Table S15: P-values between between any two collection times for each type Table. (PDF 430 kb) [file 12864_2018_5285_MOESM14_ESM.pdf]

Table S14. P-values between the Type 1 and Type 2 groups

NO	Blood	Type 1 vs Type 2 SB	D7	D28
1	Hematocrit	0.879	0.543	0.342
2	Hemoglobin	0.849	0.676	0.518
3	RBC	0.621	0.503	0.412
4	WBC	0.093	0.287	0.594
5	Platelet	0.362	0.447	0.425
6	Neutrophils	0.543	0.882	0.879
7	Eosinophils	0.171	**0.013**	0.094
8	Basophils	0.079	**0.001**	**0.011**
9	Monocytes	0.362	0.342	0.939
10	Lymphocytes	0.941	0.552	0.676
11	Albumin	0.142	0.645	0.334
12	Alkaline_phosphatase	0.567	0.790	0.592
13	SGPT	**0.033**	**0.017**	**0.013**
14	SGOT	0.169	**0.011**	**0.049**
15	CRP	**0.036**	**0.044**	**0.044**
16	Amylase	0.790	0.676	0.621
17	Bilirubin	0.359	0.938	0.730
18	Creatinine	0.401	**0.033**	0.424
19	TP	1.000	0.760	0.541
20	FBG	0.848	0.381	0.286
21	Cholesterol	0.160	0.543	0.128
22	HDL	0.181	0.148	0.283
23	LDL	0.223	0.305	0.068
24	Triglycerides	0.970	0.710	0.882

* The P-values are evaluated by the Wilcoxon rank sum test.

Table S15. P-values between any two collection times for each type

NO	Blood	Type 1 SB vs D7	Type 1 SB vs D28	Type 2 SB vs D7	Type 2 SB vs D28
1	Hematocrit	0.690	0.658	0.974	0.627
2	Hemoglobin	0.894	0.723	0.818	0.824
3	RBC	0.965	0.895	0.949	0.863
4	WBC	0.929	0.594	0.509	0.626
5	Platelet	0.929	0.791	0.797	0.930
6	Neutrophils	0.965	0.931	0.341	1.000
7	Eosinophils	0.566	0.627	0.742	0.426
8	Basophils	0.824	0.687	0.664	0.929
9	Monocytes	0.157	0.658	0.622	0.215
10	Lymphocytes	1.000	1.000	0.478	1.000
11	Albumin	0.784	0.129	0.593	**0.042**
12	Alkaline_phosphatase	0.757	0.965	0.921	1.000
13	SGPT	0.930	0.930	0.792	0.757
14	SGOT	0.592	0.929	0.446	0.756
15	CRP	0.626	0.894	0.669	0.477
16	Amylase	0.965	0.859	0.818	0.930
17	Bilirubin	0.894	0.657	0.508	0.473
18	Creatinine	0.373	0.722	0.339	1.000
19	TP	0.824	0.535	0.816	0.425
20	FBG	0.352	0.859	0.197	0.250
21	Cholesterol	0.863	0.724	0.921	0.791
22	HDL	0.894	0.690	0.869	0.626
23	LDL	0.860	0.724	0.599	0.930
24	Triglycerides	0.895	1.000	0.922	0.965
