# Supplementary material for: Gut microbiome changes in overweight male adults following bowel preparation
Source: BMC Genomics. 2018 Dec 31;19(Suppl 10):904. doi: 10.1186/s12864-018-5285-6 (PMC6311932; doi:10.1186/s12864-018-5285-6)
Supplement: Supplementary file 16 — Table S17. Participant’s lifestyle. (PDF 89 kb) [file 12864_2018_5285_MOESM16_ESM.pdf]

Table S17. Participants' lifestyle

ID	Age	BMI (kg/m^2)	Fecal Type	Occupation	Smoking	Betel nut
S07	53	26.6	Type 1	Researcher	No	No
S13	33	29.2	Type 1	Engineer	No	No
S16	37	34.2	Type 1	Project manager	No	No
S19	41	26.9	Type 1	Manager	No	No
S20	28	30.1	Type 1	Researcher	No	No
S29	52	25.9	Type 1	Engineer	No	No
S31	45	25.7	Type 1	Researcher	No	No
S32	40	31.6	Type 1	Engineer	NA	No
S33	40	26.7	Type 1	Engineer	NA	No
S04	48	29.7	Type 2	Service industry	No	No
S05	35	33.0	Type 2	Taxi driver	Yes	Ever
S06	39	30.1	Type 2	Deliveryman	Yes	Yes
S08	36	26.8	Type 2	Engineer	Yes	No
S10	49	26.8	Type 2	Manager	Yes	No
S11	31	33.3	Type 2	NA	NA	No
S14	32	28.7	Type 2	Motorcycle technician	Yes	No
S21	47	27.5	Type 2	Elementary teacher	Yes	No
S23	44	26.7	Type 2	Salesman	No	No
S34	41	27.6	Type 2	Engineer	NA	No
S36	38	31.3	Type 2	Computer engineer	NA	No

* BMI: Evaluated at prior to bowel preparation
