# Supplementary material for: Gut microbiome changes in overweight male adults following bowel preparation
Source: BMC Genomics. 2018 Dec 31;19(Suppl 10):904. doi: 10.1186/s12864-018-5285-6 (PMC6311932; doi:10.1186/s12864-018-5285-6)
Supplement: Supplementary file 17 — Table S18. Percentage of fecal samples and mean relative abundance of SMB53, Bulleidia, and Akkermansia. (PDF 176 kb) [file 12864_2018_5285_MOESM17_ESM.pdf]

Table S18. Percentage of fecal samples and mean relative abundance of SMB53, Bulleidia, and Akkermansia

Type 1						
**Genus**	SB(N%)	D7(N%)	D28(N%)	mean_SB	mean_D7	mean_D28
SMB53	89	100	89	0.419	0.081	0.14
Bulleidia	89	67	44	0.004	0.002	0.002
Akkermansia	22	11	33	0	0.001	0.001

Type 2						
**Genus**	SB(N%)	D7(N%)	D28(N%)	mean_SB	mean_D7	mean_D28
SMB53	91	91	82	0.044	0.028	0.038
Bulleidia	55	27	55	0.001	0.001	0.002
Akkermansia	73	55	45	0.588	0.242	0.843
